# Modeling alcohol-induced neurotoxicity using human induced pluripotent stem cell-derived three-dimensional cerebral organoids

**DOI:** 10.1038/s41398-020-01029-4

**Published:** 2020-10-13

**Authors:** Thiago Arzua, Yasheng Yan, Congshan Jiang, Sarah Logan, Reilly L. Allison, Clive Wells, Suresh N. Kumar, Richard Schäfer, Xiaowen Bai

**Affiliations:** 1 Department of Cell Biology, Neurobiology & Anatomy, Medical College of Wisconsin, Milwaukee, 53226 WI USA; 2 Department of Physiology, Medical College of Wisconsin, Milwaukee, 53226 WI USA; 3 Department of Anesthesiology, Medical College of Wisconsin, Milwaukee, 53226 WI USA; 4 Department of Microbiology, Medical College of Wisconsin, Milwaukee, 53226 WI USA; 5 Department of Pathology, Children’s Research Institute Imaging Core, Neuroscience Imaging Facility, Medical College of Wisconsin, Milwaukee, 53226 WI USA; 6 Institute for Transfusion Medicine and Immunohaematology, German Red Cross Blood Donor Service Baden-Württemberg-Hessen gGmbH, Goethe University Hospital, 60438 Frankfurt am Main, Germany

**Keywords:** Molecular neuroscience, Psychiatric disorders

## Abstract

Maternal alcohol exposure during pregnancy can substantially impact the development of the fetus, causing a range of symptoms, known as fetal alcohol spectrum disorders (FASDs), such as cognitive dysfunction and psychiatric disorders, with the pathophysiology and mechanisms largely unknown. Recently developed human cerebral organoids from induced pluripotent stem cells are similar to fetal brains in the aspects of development and structure. These models allow more relevant in vitro systems to be developed for studying FASDs than animal models. Modeling binge drinking using human cerebral organoids, we sought to quantify the downstream toxic effects of alcohol (ethanol) on neural pathology phenotypes and signaling pathways within the organoids. The results revealed that alcohol exposure resulted in unhealthy organoids at cellular, subcellular, bioenergetic metabolism, and gene expression levels. Alcohol induced apoptosis on organoids. The apoptotic effects of alcohol on the organoids depended on the alcohol concentration and varied between cell types. Specifically, neurons were more vulnerable to alcohol-induced apoptosis than astrocytes. The alcohol-treated organoids exhibit ultrastructural changes such as disruption of mitochondria cristae, decreased intensity of mitochondrial matrix, and disorganized cytoskeleton. Alcohol exposure also resulted in mitochondrial dysfunction and metabolic stress in the organoids as evidenced by (1) decreased mitochondrial oxygen consumption rates being linked to basal respiration, ATP production, proton leak, maximal respiration and spare respiratory capacity, and (2) increase of non-mitochondrial respiration in alcohol-treated organoids compared with control groups. Furthermore, we found that alcohol treatment affected the expression of 199 genes out of 17,195 genes analyzed. Bioinformatic analyses showed the association of these dysregulated genes with 37 pathways related to clinically relevant pathologies such as psychiatric disorders, behavior, nervous system development and function, organismal injury and abnormalities, and cellular development. Notably, 187 of these genes are critically involved in neurodevelopment, and/or implicated in nervous system physiology and neurodegeneration. Furthermore, the identified genes are key regulators of multiple pathways linked in networks. This study extends for the first time animal models of binge drinking-related FASDs to a human model, allowing in-depth analyses of neurotoxicity at tissue, cellular, subcellular, metabolism, and gene levels. Hereby, we provide novel insights into alcohol-induced pathologic phenotypes, cell type-specific vulnerability, and affected signaling pathways and molecular networks, that can contribute to a better understanding of the developmental neurotoxic effects of binge drinking during pregnancy.

## Introduction

Alcohol consumption by pregnant women can adversely affect the developing fetus, resulting in a spectrum of abnormalities collectively known as fetal alcohol spectrum disorders (FASDs), with a prevalence of 1.1–5.0% in the USA^1,2^. The effects of prenatal alcohol (ethanol) exposure can lead to lifelong physical, behavioral, cognitive, and psychological problems. FASDs represent a major public health burden from an economic, societal, educational, family, or health or medical home perspective^3^. The severity of ethanol-induced brain dysfunction in humans varies greatly and can be placed on a continuum from subtle cognitive dysfunction and neurobehavioral deficits to obvious structural abnormalities (e.g., microcephaly and agenesis of corpus callosum)^4–7^. Approximately 90% of individuals with FASD suffered from mental problems such as depression, anxiety, suicide threats, impulsive behavior, attention deficit, hyperactivity disorder, inappropriate sexual behavior, or substance use disorder^8–11^. Such variability in neurobehavioral outcomes is associated with genetic background, alcohol consumption patterns, timing of maternal drinking in terms of gestational age, and nutritional intake^6^. For instance, it was shown that genetic polymorphisms in the alcohol dehydrogenase 1B (ADH1B) gene, a gene responsible for alcohol metabolism, can lead to an increase of vulnerability to FASDs^12^. Furthermore, binge drinking during pregnancy was associated with lower academic scores and behavioral disorders^13,14^, while continuous drinking throughout the pregnancy can cause fetal alcohol syndrome (FAS), which is the most severe type of FASDs and exhibits both physical and neurobehavioral problems^15^. Binge drinking is defined as a pattern of alcohol consumption that elevates blood alcohol concentration (BAC) to 80 mg/dL or above. For women, this usually occurs when consuming 4 or more standard drinks within 2 hours (h). One standard drink is equal to ~330 mL regular beer or 150 mL wine^16^. It was reported that 1 in 33 pregnant women had binge drinking episodes^17,18^. Epidemiological studies in humans have confirmed that children of binge-drinking mothers exhibited severe cognitive and behavioral deficits such as low academic performance, hyperactivity, impulsivity, learning disability, and attention deficiency^19,20^.

Despite well-known adverse consequences, 1 in 10 pregnant women reported that they consumed alcohol during pregnancy^17^. Different sociocultural factors, including unplanned pregnancies^21^ as well as alcohol abuse disorder (AAD), not only make it difficult to prevent drinking with education alone, but it also means that the actual prevalence of FASDs might be higher than estimated^22,23^. As a result, in the USA, approximately $5.4 billion per year is spent on treating FASDs^24^. This information suggests that FASDs are concerning on a societal level. Thus, understanding the cause of FASDs including dose-response of human brain cells to the ethanol and the underlying mechanisms of neurobehavioral outcomes is urgently needed in order to develop effective prevention and early intervention programs.

There are pragmatic and ethical barriers to conduct direct research on human subjects, particularly on brain samples. In addition, there is insufficient data from human models that adequately recapitulate the human developing brain. Animal models have played a major role in studying FASDs for decades and have greatly facilitated an understanding of how ethanol affects the developing nervous system^25–27^. Observations in animals exposed to ethanol at different points during gestation closely mimicked the pathologies, cognition dysfunction and abnormal behavior observed in patients with FASDs^28–31^. These studies have provided valuable insight into the underlying cellular and molecular mechanisms, such as cell apoptosis, inflammation, and decreased neuronal plasticity^6,32^. However, acknowledging inherent species-specific differences, considerable questions have emerged regarding the translatability of such animal-based research since there are many differences in physiology, genetics, and developmental patterns between human and animal brains^33–36^. Early studies using human tissues were limited to either isolation from fetal brains directly, or to differentiation from human embryonic stem cells^37,38^. With ethical and practical barriers to more refined studies in these models, researchers turned to human-induced pluripotent stem cells (iPSCs) and iPSC-derived neurospheres to investigate the neurotoxic effects of ethanol. iPSCs can be reprogrammed from somatic cells, e.g., fibroblasts, and urine and blood cells. They replicate indefinitely and can be differentiated into every cell type of the body as shown in our previous and others’ studies^39–42^. Neurospheres are three dimensional (3D) aggregates of neural progenitor cells that are useful to evaluate phenotypes related to early differentiation and proliferation^43^. For instance, Donadoni et al exposed a mixed culture of neurospheres and primary human fetal neurons to increasing concentrations (0, 10, 25, 50, and 75 mM) of ethanol for 6, 24, and 48 h^44^. Their results showed that these progenitor cells and neurospheres were sensitive to ethanol, undergoing apoptosis through alternative splicing of Mcl-1L. This was a key study in replicating findings of programmed cell death in human neural progenitor cells. However, neurospheres largely lack specific structure, or cell diversity when compared to more complex models such as 3D cerebral organoids. These organoids, informally depicted “mini brains”, are also derived from human iPSCs and they are a promising next step in the toolbox of models available to study FASDs with a more clinically relevant high throughput cell culture system^36,40^.

In 2013, Dr. Knoblich’s lab developed an in vitro system of generating cerebral organoids by growing human iPSCs in Matrigel, a scaffold resembling the extracellular matrix, which allowed the cells to differentiate into cellular layers similar to those of real developing brains^36^. Recent data from us and others additionally support that cerebral organoids structurally and developmentally recapitulate fetal brains with higher fidelity than the currently used 2D brain cell models^36,40,45–51^. Cerebral organoids have been used for modeling several human developmental brain diseases (e.g., microcephaly, autism, and Zika virus infection), and the findings have provided novel insights into these diseases’ mechanisms and treatment^36,40,52–60^. In addition, there has been only one study of using a ≤1-month cerebral organoid model to detect the toxic effect of chronic ethanol exposure (14 days) on the early stage (days 10 to 24) developed organoids. The data showed that with ethanol exposure, the brain organoids displayed attenuated neurite outgrowth and skewed neural maturation^61^. The study also showed an increase in mRNA expression of caspase 3, measured by RT-qPCR, but no evidence of activation of caspase 3 at the protein level for the confirmation of apoptosis. Lastly, the binge drinking-induced pathology and molecular changes on developing human brain tissues is largely unknown.

The aim of this study was to conduct for the first time the investigation on 2-month-old human iPSC-derived cerebral organoids and dissect and answer the following questions that we do not know so far on human brains: (1) whether binge drinking-like alcohol (ethanol) exposure induces apoptosis, ultrastructure changes, and mitochondrial dysfunction, (2) what is the lowest ethanol concentration that can trigger cell death, and (3) which brain cells (neurons vs. astrocytes) are more vulnerable to apoptotic action of ethanol. We further performed transcriptomic studies using microarray assays and conducted bioinformatics analyses to identify signaling pathways associated with ethanol-induced brain injury.

## Materials and methods

### Human iPSC culture and expansion

**Fig. 1:**
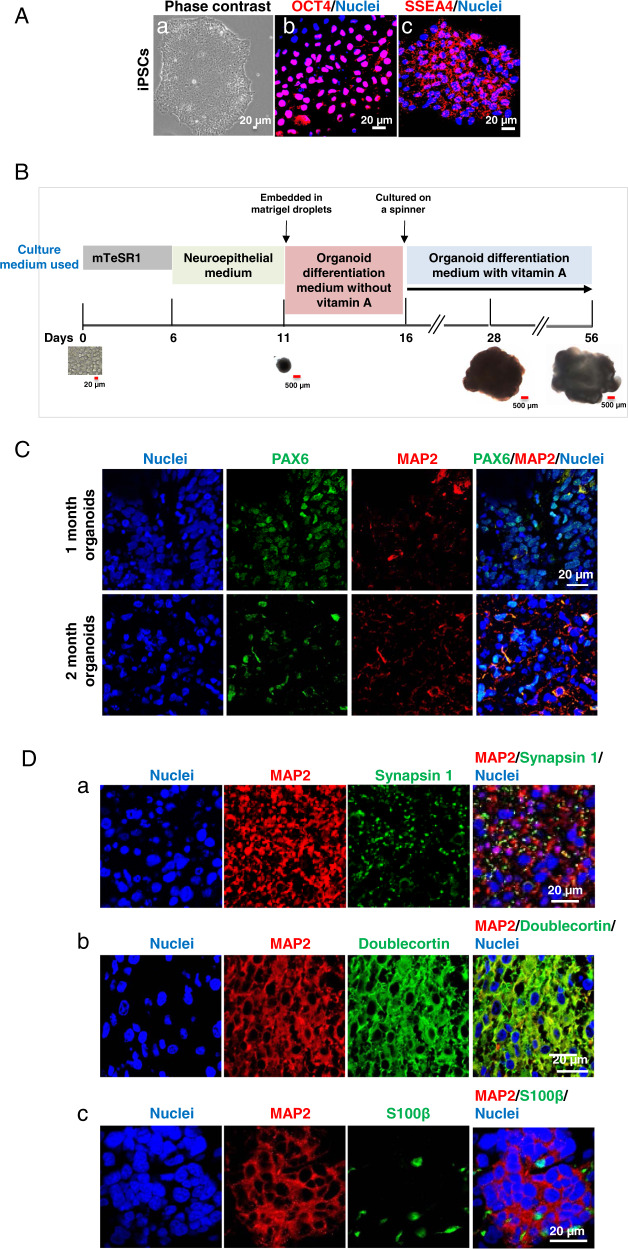

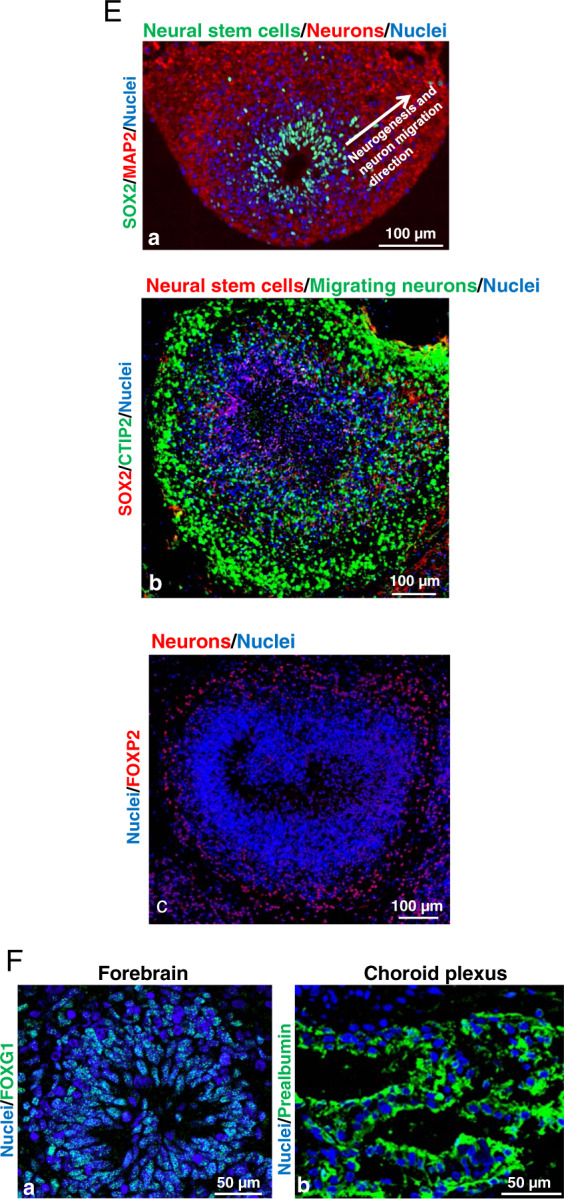
Characterization of human-induced pluripotent stem cells (iPSCs) and iPSC-derived cerebral organoids over the two-month differentiation process from iPSCs to cerebral organoids. **A** Human iPSCs grew as colonies in the culture and expressed pluripotent stem cell markers octamer binding transcription factor 4 (OCT4) and stage-specific embryonic antigen-4 (SSEA4). The phase-contrast image shows the colonies of iPSCs maintained in mTeSR1 stem cell culture medium (a). Confocal images of the cells with immunofluorescence staining demonstrate that iPSCs colonies expressed OCT4 (red) in the nuclei (b) and SSEA4 (red) in cell member (c). Nuclei were stained with Hoechst 33342 in blue. Scale bar = 20 µm. **B** The schematic depicts the procedure for the generation of organoids from iPSCs by the use of chemically defined medium and plating strategies. Singularized iPSCs cultured in stem cell medium mTeSR1 aggregated into embryoid bodies in ultra-low attachment plates (day 0 to day 6), begun to differentiate into neuroepithelial tissue, and resulted in the formation of cerebral organoids following Matrigel™ embedding at day 11. The organoids grow bigger over time in the culture. Scale bar = 20 or 500 μm. **C** Cerebral organoids develop over time in the culture. Immunofluorescent staining marked the expression of the paired box protein 6 (PAX6) in neural epithelial progenitor cells (green) in 1-month organoids, which was markedly reduced in 2-month organoids. Microtubule-associated protein 2 (MAP2)-positive neurons (red) were expressed in 1-month cerebral organoids, but more fluorescence signals were observed in 2-month organoids. Blue are cell nuclei. Scale bar = 20 µm. **D** Cerebral organoids contain neurons and astrocytes. In 2-month old, immunofluorescent staining for pre-synapse marker synapsin I (green; distributed in a punctuate pattern) shows a vast number of synapses between MAP2-positive neurons (red) (a). The neurons (red) were immature as evidenced by the expression of doublecortin (green) (b). Astrocytes, as indicated by the S100 calcium-binding protein β (S100β) expression (green) were also present with neurons (red) in the organoids (c). Nuclei were stained in blue. Scale bar = 20 μm. **E** Two-month cerebral organoids display well-organized elaborate cellular laminar organization and architectures (neural stem cells and neurons expressing different cortex layer neuron markers are located in the different layers of the organoid tissues). SOX2-positive neural stem cells (green) are located on the apical side and neurons (red) are located on the basal side (a), suggesting that neural stem cell-derived neurons migrate from the apical toward the basal side. The white arrow indicates the migration direction of differentiated neurons. Neurons in cerebral organoids also expressed deep-layer cortical neuron markers CTIP2 (b, green) and FOXP2 (c, red). Blue represents cell nuclei. Scale bar = 100 μm. **F** Cerebral organoids display FOXG2-positive (green, a) forebrain and prealbumin-positive choroid plexus-like (green, b) brain regions. Blue represents cell nuclei. Scale bar = 50 μm.

**Fig. 2:**
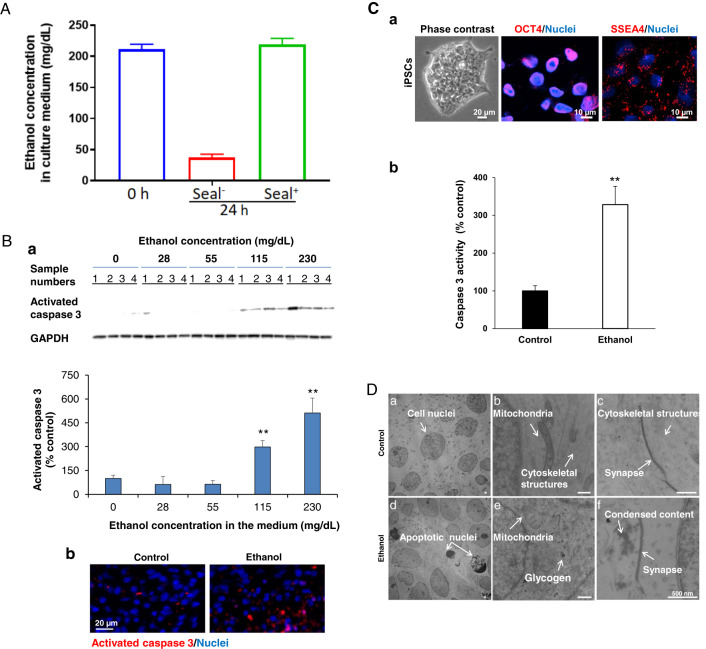
Characterization of ethanol-induced apoptosis and ultrastructure changes in cerebral organoids derived from human iPSC line 1 (B and D) and line 2 (C). **A** Analysis of ethanol concentration in culture medium over 24-h ethanol (230 mg/dL) exposure using Alcohol Assay Kit. The culture dishes were sealed with Parafilm™ during the ethanol treatment to prevent ethanol evaporation. Alcohol maintained the same concentration in culture medium over 24-h ethanol exposure. However, ethanol concentration dramatically decreased in the non-sealing dishes (*n* = 3). **B** Ethanol exposure dose-dependently induces apoptosis in iPSC-derived cerebral organoids. Two-month human iPSC line 1-derived cerebral organoids were treated with increasing concentrations of ethanol (0, 28, 44, 115, and 230 mg/dL). Western blot assay showed that ethanol dose-dependently increased the expression of activated caspase 3 (an apoptotic cell marker) in iPSC-derived organoids compared to the control group (**B**-a). Glyceraldehyde 3-phosphate dehydrogenase (GAPDH) was used as an internal housekeeping gene for normalization of activated caspase 3 expression data in the Western blot assay. Data are presented as mean ± standard error of the mean (SEM), *n* = 4, **P < 0.01 vs. non-ethanol treatment control. Immunofluorescence staining confirms that ethanol (230 mg/dL) treatment increased activated caspase 3-positive apoptotic cells (red) in the organoids. Nuclei were stained in blue (**B**-b). Scale bar = 20 μm. **C** Human iPSC line 2 grew as colonies in the culture and expressed pluripotent stem cell markers OCT4 (red) and SSEA4 (red). Nuclei were stained with Hoechst 33342 in blue. Scale bar = 20 or 10 µm (**a**). Ethanol (230 mg/mL, 6 h) induces apoptosis in human iPSC line 2-derived cerebral organoids (b). *n* = 4, *P < 0.05 vs. non-ethanol treatment control. **D** Electron microscopy images of apoptosis, cellular and subcellular alterations of cells in control (**D**-a to c), and ethanol (230 mg/mL, 6 h)-treated cerebral organoids (**D**-d to f). In the control organoids, cells appeared healthy with normal nuclei (**D**-a), normal mitochondria (**D**-b), and well-organized cytosolic structure. Cytoskeletal structures appeared to be present and looking normal (**D**-b and c) around nuclei and synapse (**D**-c). Well organized and homogenous cellular content was also observed. In the ethanol-treated organoids, apoptotic dark nuclei, condensed and fragmented chromatin appeared in the shrunken apoptotic cells. Formation of classic apoptotic ‘half-moon’ nuclear morphology bodies was seen in some cells (**D**-d). Abnormal mitochondria with less dense matrix and disrupted cristae were commonly observed together with abundant glycogen compared with control cells (**D**-e). There appeared to be disruption of the cytoskeleton with components not being visible within cells and the presence of condensed content around synapse (**D**-f). Scale bar = 500 nm.

**Fig. 3:**
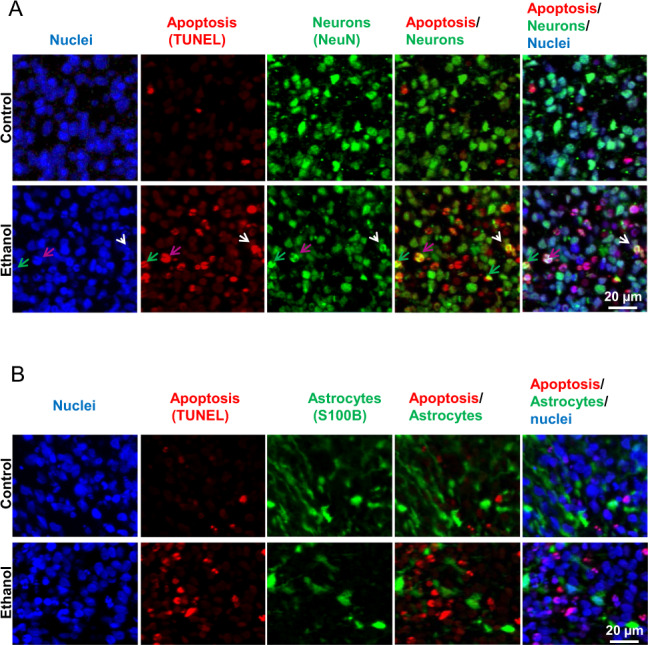
Ethanol (230 mg/mL, 6 h) induces apoptosis in neurons but not astrocytes in 2-month cerebral organoids from iPSC line 1. **A** NeuN and TUNEL co-stained fluorescent images of cerebral treated with or without 50 mM ethanol for 6 h. In order to identify whether neurons in organoids undergo apoptosis following ethanol exposure, the brain section was co-stained with TUNEL (detection of the DNA fragmentation caused by apoptotic signaling cascades) and neuronal nuclear antigen (NeuN; a neuron marker). Cell nuclei were stained with Hoechst 33342. Blue, green, and red represent cell nuclei, NeuN, and TUNEL signals, respectively. These images include either individual channel or overlaid images, showing that ethanol treatment induces apoptosis in neurons as evidenced by co-localized TUNEL and NeuN signals in the same neurons. Three representative NeuN and TUNEL double-positive apoptotic neurons are indicated by pink, white, and green arrows. Scale bar = 20 µm. **B** Fluorescence images S100β (an astrocyte marker) and TUNEL co-stained organoids. Blue, green, and red represent cell nuclei, S100β, and TUNEL, respectively. The images showed that TUNEL and S100β double-positive apoptotic astrocytes were not increased in ethanol-treated organoids, indicating that ethanol does not cause astrocyte apoptosis. Scale bar = 20 µm.

**Fig. 4:**
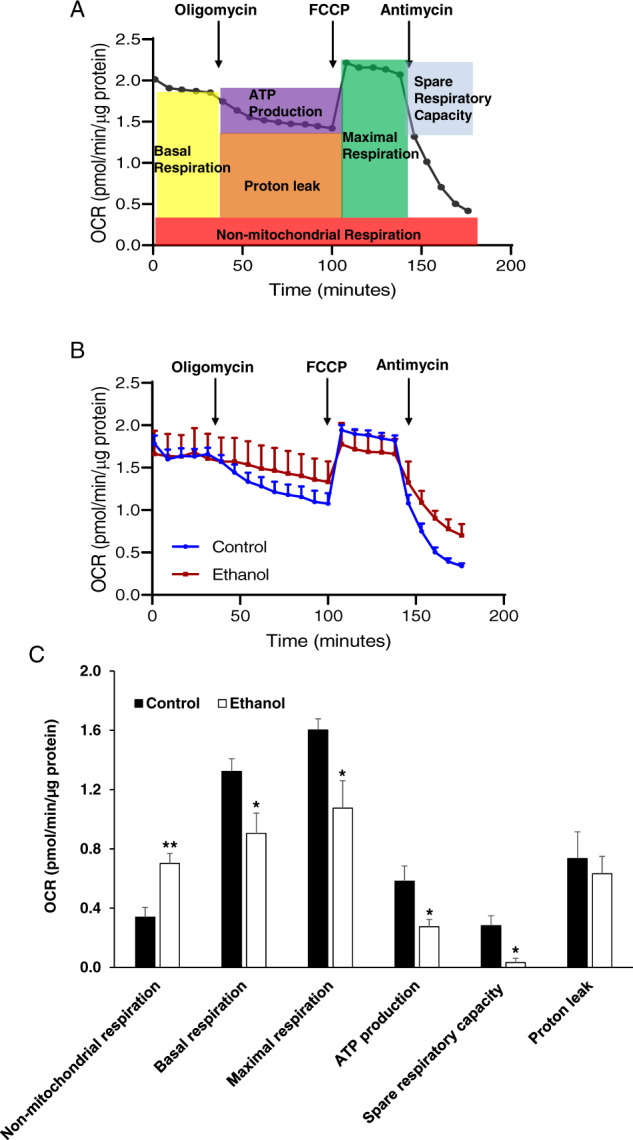
The effect of ethanol (230 mg/mL, 6 h) on the mitochondrial respiratory capacity of iPSC line 1-derived cerebral organoids. **A** The diagram depicts the trace of oxygen consumption rate (OCR) on cerebral organoids after sequential administration of 10 µM oligomycin [adenosine triphosphate (ATP) synthase inhibitor], 10 µM carbonyl cyanide-4-(trifluoromethoxy)phenylhydrazone (FCCP; uncoupler of oxidative phosphorylation in mitochondria), and 10 µM antimycin A (electron transport chain blocker by inhibiting complex III). The profiles of fundamental parameters of OCR including mitochondrial and non-mitochondrial function measured are basal respiration, ATP production, proton leak, maximal respiration, spare respiratory capacity, and non-mitochondrial respiration that were marked with a different color in the schematic. **B** OCR traces of control and 230 mg/dL ethanol-treated cerebral organoids for 6 h in response to oligomycin, FCCP, and antimycin A. **C** OCR parameters (basal respiration, ATP production, maximal respiration, and spare respiratory capacity) representing mitochondrial function in ethanol-treated cerebral organoids were significantly reduced while non-mitochondrial respiration was increased following ethanol exposure. *n* = 4, **P < 0.01, *P < 0.05 vs. control organoids.

**Fig. 5:**
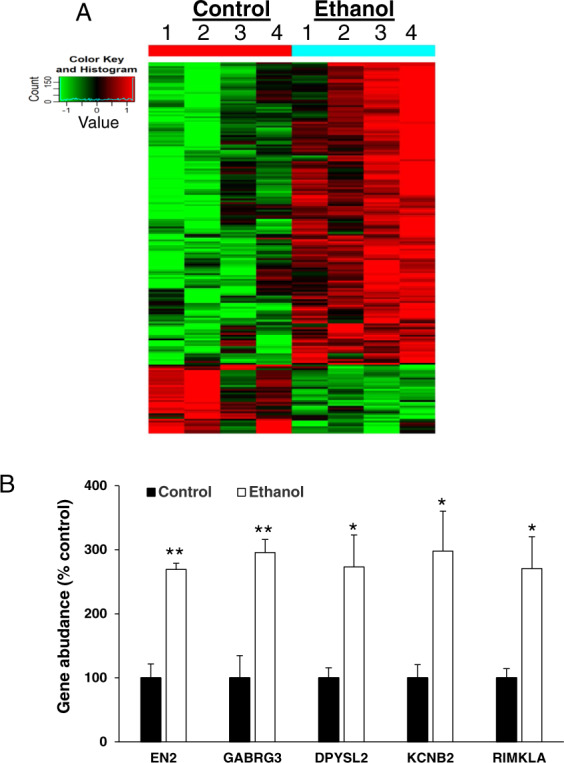
Ethanol-induced abnormal gene expression profiles in iPSC-derived cerebral organoids. **A** Differentially expressed genes between control and ethanol (230 mg/mL, 6 h)-treated cerebral organoids from iPSC line 1 are displayed as a heatmap. Green indicates relative lower expression levels of the genes and red indicates relative higher expression levels compared between control and ethanol groups (*n* = 4; *P* < 0.05). Supplemental Table 1 includes abnormally expressed individual gene profiles. Supplemental Table 2 describes the reported roles of dysregulated genes in neurodevelopment, nervous system physiology, and neurodegeneration. **B** Reverse transcription-quantitative polymerase chain reaction (RT-qPCR) validation of five representative altered genes from array data. *n* = 4; **P < 0.01, *P < 0.05 vs. control group. EN2 engrailed homeobox 2, GABRG3 gamma-aminobutyric acid type A receptor gamma3 subunit, DPYSL2 dihydropyrimidinase like 2, KCNB2 potassium voltage-gated channel subfamily B member 2, RIMKLA ribosomal modification protein rimK like family member A.

**Fig. 6:**
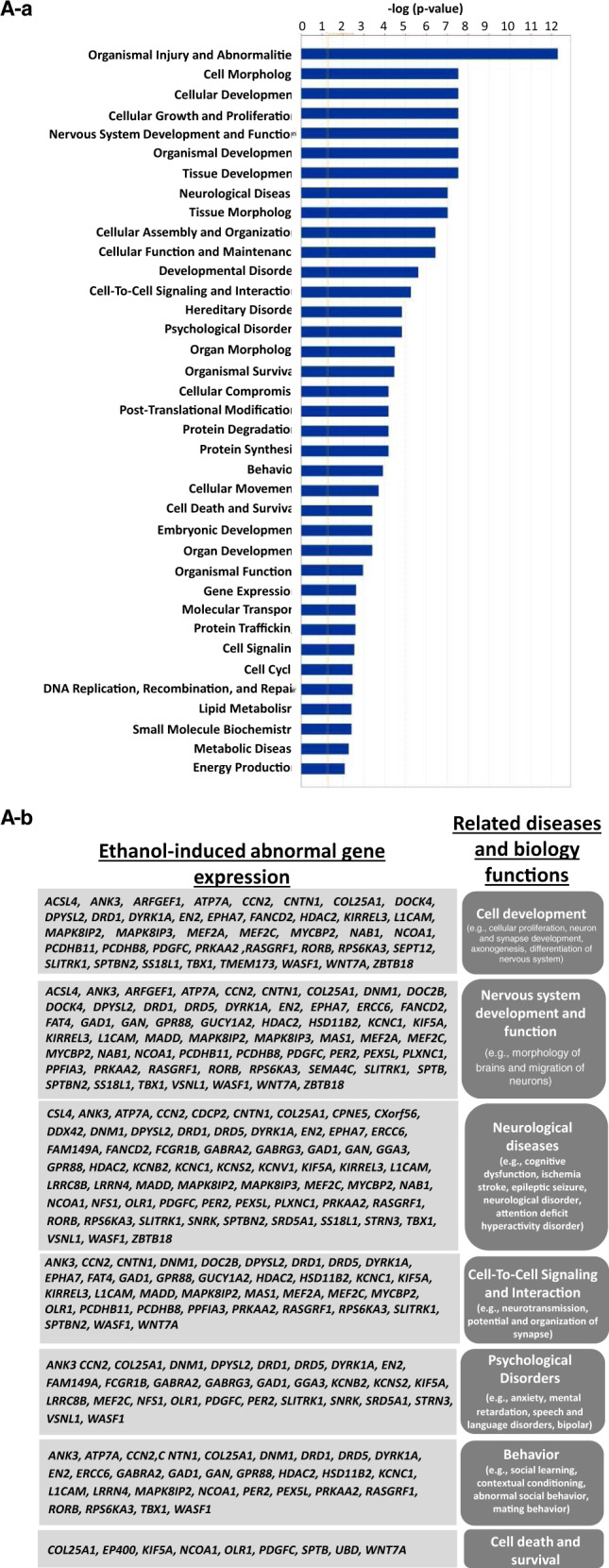

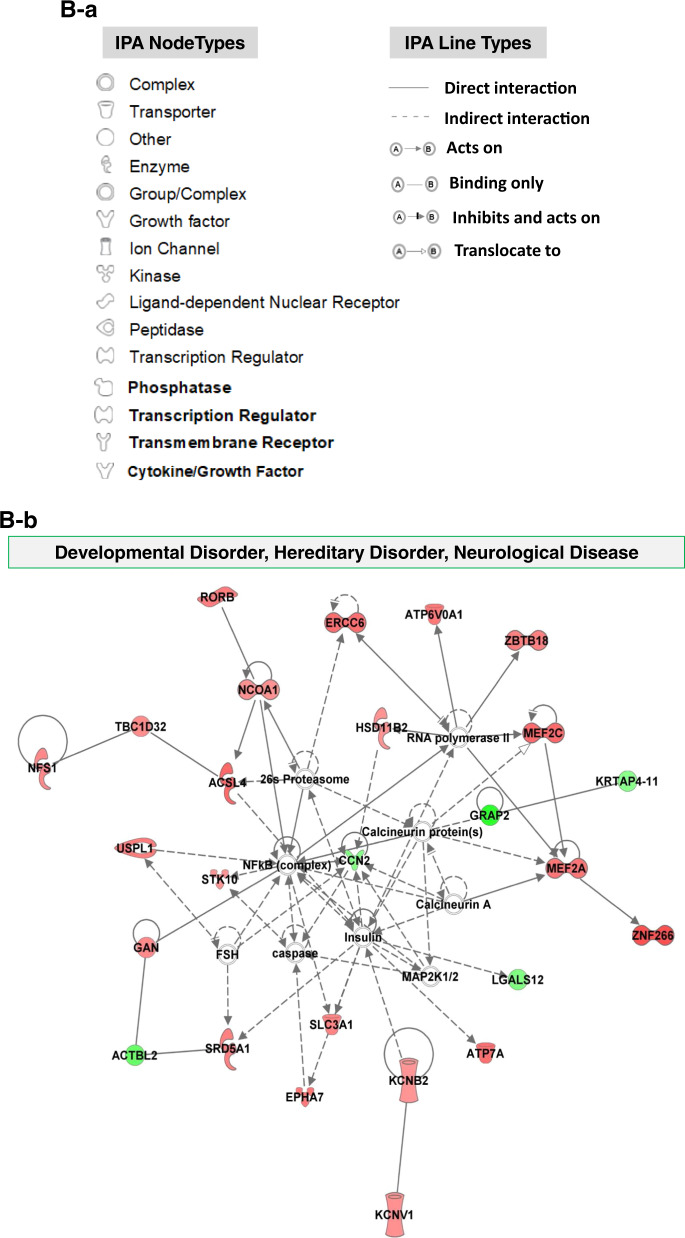

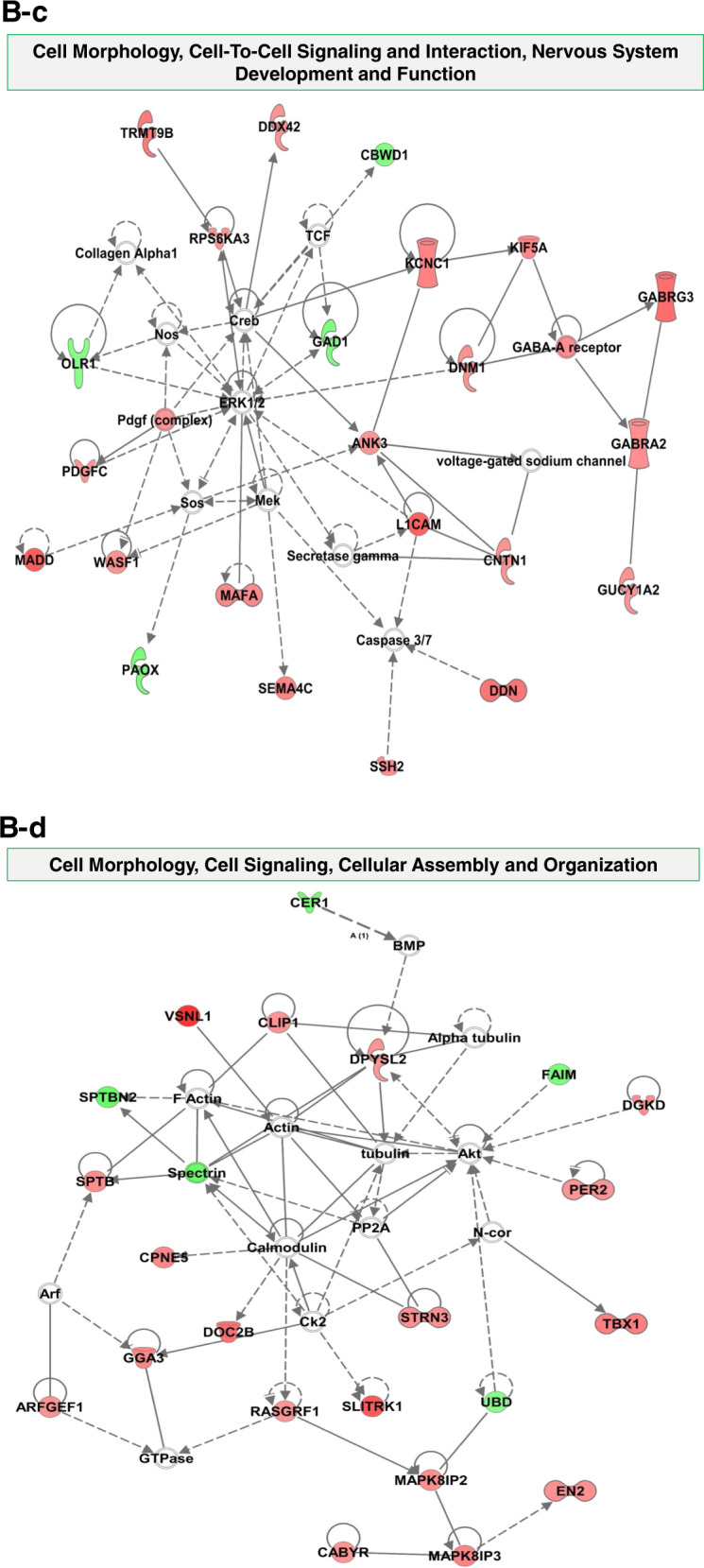
Bioinformatics analysis of ethanol (230 mg/mL, 6 h)-induced altered gene-related signaling and networks in 2-month cerebral organoids from iPSC line 1 using Ingenuity Pathway Analysis (IPA) software. **A** Ethanol-induced abnormally expressed gene-related 37 diseases and biological functions (a), and representative 7 signaling networks (right column) that are associated with were associated with the development and neurodegenerative phenotypes and the corresponding dysregulated genes conferred by ethanol exposure (left column) (b). Supplemental Table 3 includes all abnormally expressed genes related to 37 diseases and function signaling shown in Fig. 6A-a. **B** Exploring the mechanistic networks of the ethanol-induced dysregulated genes by network analysis using IPA tools. (**B**-a) Defining various nodes and lines depicted in Figs. **B**-b to d. Each symbol represents one individual gene category such as enzyme and ion channel. Solid and dotted lines show a direct and indirect connection between genes. (**B**-b to c) The main associated functions of each network are as follows: developmental disorder, hereditary disorder, neurological disease (b); cell morphology, cell-to-cell Signaling, and interaction, nervous system development and function (c); and cell morphology, cell signaling, cellular assembly and organization (d). Gene names are shown on the network map. Green symbols indicate downregulation and red indicate upregulated genes in ethanol-treated 2-month cerebral organoids relative to control organoids. The abbreviations of the genes were defined in Supplemental Table 1.

Experiments utilized two donor-derived iPSC lines and were approved by the Medical College of Wisconsin Institutional Review Board. iPSC lines 1 and 2 were kindly provided by in the laboratories of Dr. Douglas Melton (Department of Stem Cell and Regenerative Biology, Harvard University) and Dr. Li-Huei Tsai (Department of Brain and Cognitive Sciences, Massachusetts Institute of Technology), respectively. The iPSCs were reprogrammed from fibroblasts as described previously^16,41,42^. iPSCs were maintained on Matrigel™ (Corning Inc, Corning, NY, USA)-coated Petri dishes in mTeSR1 medium (feeder-free cell culture medium for iPSCs; STEMCELL Technologies, Vancouver, BC, Canada) supplemented with 1% penicillin/streptomycin (Thermo Fisher Scientific, Waltham, MA, USA) under hypoxic conditions (4% O_2_) at 37 °C. The culture media was changed daily. Upon reaching 80% confluency, iPSCs were gently dissociated and passaged at a ratio of 1:6 using Versene (Thermo Fisher Scientific). The iPSC line 1 was used in the studies described in Figs. 1, 2a, b, d, 3 to 6, and iPSC line 2 was used in the caspase 3 activity assay shown in Fig. 2c. MycoAlert® Mycoplasma Detection Kit (Lonza) was used for analyzing whether the iPSC culture was contaminated with mycoplasma. The detection revealed that our iPSC culture contained no mycoplasma contamination.

### 3D cerebral organoid generation from iPSCs

The protocol we used for the generation of cerebral organoids was developed by Lancaster et al.^62^. In brief, we generated organoids from iPSCs by use of chemically defined medium and plating strategies (Fig. 1) as we and other described previously^51,62^ and in detail in Supplemental Materials and Methods. Singularized iPSCs in mTeSR1 media were added to each well of 96-well plates and cultured in an incubator (5% CO_2_, 21% O_2_) at 37 °C. On day 6, formed embryoid bodies were transferred to 24-well plates and cultured for 5 days. On day 11, the tissues were embedded in Matrigel droplets and plated on 100 mm plates in organoid differentiation media for 5 days. Plates were transferred to a spinner platform (horizontal shaker) on day 16 for long-term culture. Cerebral organoids were cultured up until 2 months. iPSC colonies, embryoid bodies, neuroepithelial tissues, and cerebral organoids were examined daily under the microscope and imaged under bright field by an EVOS FL Auto microscope (Thermo Fisher Scientific). All experiments below were from the 0, 1, or/and 2-month-old cerebral organoids. Each sample (*n* = 4) per group was pooled from three organoids for protein and RNA assays. Each sample was generated using different preparation of iPSCs cultured in different dishes.

### Charization of iPSCs, cerebral organoids, and apoptosis using immunostaining

The detailed immunostaining procedure for iPSC culture and cerebral organoid tissue sectioning was described in Supplemental Materials and Methods, and in our publications^41,42,51,63^. In brief, iPSC cultured on the Matrigel™-coated coverslips were sequentially fixed with 4% paraformaldehyde. The cells were then stained with the following primary antibodies at 4 °C overnight: specific embryonic antigen 4 (SSEA4: a pluripotent stem cell marker) and octamer-binding transcription factor 4 (OCT4: a pluripotent stem cell marker). After washing with phosphate-buffered saline (PBS) (Thermo Fisher Scientific) twice, the cells were incubated with a secondary antibody Alexa Fluor 594 nanometer conjugated immunoglobulin G (IgG) (Thermo Fisher Scientific). Hoechst 33342 (Thermo Fisher Scientific) was used to stain cell nuclei. Samples were imaged by laser-scanning confocal microscopy (Nikon Eclipse TE2000-U, Nikon, Minato, Tokyo, Japan).

Fixed cerebral organoid tissue sections with 4-µm thickness were stained with the following primary antibodies: paired box protein 6 (PAX6, a neural stem cell marker^64,65^), microtubule-associated protein 2 (MAP2: a neuron-specific marker), synapsin1 (a synapse marker), S100 calcium-binding protein β (S100β: an astrocyte marker), doublecortin (an immature and migrating neuron marker^66,67^), SRY-Box Transcription Factor 2 (SOX2: a neural stem cell marker^68^), Forkhead box G1 (FOXG1: a forebrain marker^56,65,69,70^), prealbumin (also called TTR: a choroid plexus marker^65,71^), COUP-TF-interacting protein 2 (CTIP2: a V/VI cortical layer migrating neuron marker^65,69,70^), and FOXP2 (a VI cortical layer neuron marker^72,73^), and cleaved (or activated) caspase 3 (an apoptosis marker). Slides were imaged by Olympus slide scanners (Olympus, Shinjuku City, Tokyo, Japan).

### Caspase 3 activity assay

Caspase 3 activity assay were analyzed using Caspase 3 Colorimetric Assay Kit (Sigma Aldrich, MO, USA). In brief, the caspase 3 activity in the cell lysate was analyzed by administration of the caspase 3 substrate acetyl-Asp-Glu-Val-Asp p-nitroanilide (Ac-DECD-pNA) and incubated for 90 min. The concentration of the reaction product pNA was detected at 405 nm and caspase 3 enzyme activity was calculated using the following formula:

$${\mathrm{Caspase}}\,3\,{\mathrm{activity}}\,({\upmu}{\mathrm{mol}}\,{\mathrm{pNA}}/{\mathrm{minutes}}/{\mathrm{mL}}) = \frac{{{\upmu}{\mathrm{mol}}\,{\mathrm{pNA}}\times{\mathrm{dilution}}\,{\mathrm{factor}}}}{{{\mathrm{Reaction}}\,{\mathrm{time}}\times{\mathrm{total}}\,{\mathrm{volume}}}}$$

### Analysis of cell vulnerability to ethanol treatment using immunofluorescence and TUNEL staining

To identify which types of brain cells (neurons vs. astrocytes) undergo apoptosis following ethanol exposure, the 2-month fixed cerebral organoid sections were subjected to sequential immunofluorescence and TUNEL staining as described in Supplemental Methods and Materials. The primary antibodies against neuronal nuclear antigen (NeuN: a neuron marker) and S100β (an astrocyte marker], and the corresponding secondary antibodies of Alexa Fluor 488-conjugated IgG were used for immunofluorescence staining. To assess cell death in the cerebral organoid, TUNEL (terminal deoxynucleotidyl transferase-mediated deoxyuridine triphosphate in situ nick end labeling)staining was performed using in situ Cell Death kit, TMR red (Roche Applied Bio Sciences, Indianapolis, IN) following the instruction from the manufacturer and as we previously described^74–76^. Slides were imaged using whole slide scanners (Olympus, USA).

### Ethanol exposure and ethanol concentration assay

We treated 2-month cerebral organoids with different concentrations of ethanol (50, 25, 12.5, 6.25, and 0 mM, equivalent to 230, 115, 55, 28 and 0 mg/dL) for 6 h by mixing ethanol (Sigma-Aldrich) into 15 mL of culture medium and adding it to the cell culture plate. Binge drinking can bring BAC to 80 mg/dL or above. The ethanol concentration of 230, 115, 55, 28 mg/dL can be resulted from appropriately 5 to 10, 3 to 5, 1 to 2, 1 standard drinks, respectively, depending on body weight. The ethanol doses were selected to be equivalent to or below BAC from binge drinkers (>4 standard drinks) in order to find the lowest ethanol concentration that can trigger apoptosis in cerebral organoids. Ethanol-containing medium was prepared freshly before use. Both ethanol and control culture dishes were sealed with Parafilm™ during the ethanol treatment to prevent ethanol evaporation. The pH in the medium was not affected by the sealing with parafilm. The culture system was used previously by others in relevant cell culture experiments^77–79^. Ethanol concentrations in culture media were monitored using Ethanol Assay Kit (Colorimetric) (Cell Biolabs, INC, San Diego, CA, USA) following the product manual. As shown in the Fig. 2A-a, ethanol exposure for 6 h exhibited the dose-dependent neuroapoptotic effect on 2-month organoids. The 230 mg/mL ethanol-treated organoids displayed the most cell injury. Thus, we used the condition of 230 mg/mL ethanol exposure for 6 h in other experiments and the related data shown in Figs. 2B-b, 2C, 3 to 6.

### Electron microscopy analysis

Cerebral organoids were fixed with 2% glutaraldehyde in 0.1 M sodium cacodylate buffer pH 7.4 at 4 °C, washed 3 × 15 minutes with cold 0.1 M buffer then postfixed for 2 h with potassium ferrocyanide-reduced 1% osmium tetroxide on ice. Organoids were washed 2 × 15 minutes with distilled water, dehydrated through graded methanol solutions to anhydrous 100% methanol than via acetonitrile into epoxy resin (EMBed 812, Electron Microscopy Sciences, Hatfield, PA, USA). Organoids were polymerized overnight at 70 °C. Thereafter, 60 nm thick sections were cut and stained with uranyl acetate and lead citrate. Sections were imaged using an H600 Electron Microscope (Hitachi, Chiyoda, Tokyo, Japan).

### Protein quantification

Protein quantification for bioenergetics analysis was conducted using the DC^TM^ Protein Assay kit (Bio-Rad, Hercules, CA, USA) according to the manufacturer’s instructions. The absorbance at 750 nm of each sample and known standards of bovine serum albumin (BSA) was analyzed using a Microplate Reader (Bio Tek). The standard curve of absorbance vs. concentration of known standards of BSA (Bio-Rad) was plotted and then used for determining the concentration of unknown protein samples based on their absorbance.

### Western blot

Cerebral organoids were lysed on ice with radioimmunoprecipitation assay (RIPA) lysis buffer (Cell Signaling Technology, Danvers, MA, USA) in the presence of phenylmethylsulfonyl fluoride (Sigma-Aldrich) and phosphatase inhibitor tablets (Roche, Mannheim, Germany). The protein samples were boiled for 5 min at 97 °C. The total protein of 15 μg was loaded for Western blot assay. Blots were incubated with primary antibodies rabbit anti activated caspase 3 or rabbit anti-GAPDH (Cell Signaling) overnight on a rocker at 4 °C. The primary antibodies were then washed out with Tris-buffered saline including 0.1% Tween 20. Subsequently, the membranes were incubated with secondary antibodies conjugated to horseradish peroxidase (Cell Signaling) for one hour at room temperature. The labeled proteins were detected with ECL Prime Western Blotting Reagents (GE Healthcare, Chicago, IL, USA) and imaged using a ChemiDoc MP imaging system (Bio-Rad) as previously described^75,80^. The optical densities of the proteins were quantified. The activated caspase 3 abundance in the organoids was normalized to glyceraldehyde 3-phosphate dehydrogenase (GAPDH) (Cell Signaling) and presented as a percentage of the non-ethanol control organoids.

### Mitochondrial bioenergetic analysis

Agilent Seahorse XFe96 Spheroid Microplates (Agilent Technologies, Santa Clara, CA) were used for the energetic analysis of live cerebral organoids following the guideline from the manual. Oxygen consumption and extracellular acidification rates generated by the tissues between 200 and 500 μm are within the dynamic range of the oxygen and pH sensors. Using spheroids larger than 500 μm is not recommended due to the size limitations of the measurement chamber. Two-month control and ethanol-treated cerebral organoids with a diameter of around 6 mm were sliced into 10 pieces and two randomly selected pieces (as technical duplicate) from each organoid were plated to two polylysine (100 µg/mL; Sigma-Aldrich)-coated wells, respectively in Agilent Seahorse XFe96 Spheroid Microplate. The assay medium contained unbuffered RPMI supplemented with 100 nM insulin (Sigma-Aldrich) and 11.1 mM glucose (for mitochondrial oxidation assay) or no glucose (for glycolysis assay). Mitochondrial oxidation was evaluated by analysis of oxygen consumption rate (OCR, pmol/min/µg protein) using an XFe96 Extracellular Flux Analyzer (Agilent, Santa Clara, CA, USA), respectively. For the mitochondrial oxidation assay, OCRs were obtained from the slope of change in oxygen over time. After measurements of the baseline OCR, OCRs were analyzed by sequential automatic injections of the following substrates and inhibitors with a final concentration of 10 μM oligomycin [adenosine triphosphate (ATP) synthase inhibitor] (Sigma-Aldrich), 10 μM carbonyl cyanide p-(trifluoromethoxy) phenylhydrazone (FCCP, uncoupler of oxidative phosphorylation in mitochondria) (Sigma-Aldrich), and 10 μM antimycin A (electron transport chain complex III blocker) (Sigma-Aldrich). Each OCR parameter we assessed was calculated as below: (1) basal respiration: The last rate measurement before oligomycin injection minus the non-mitochondrial respiration rate (the minimum rate measurement after antimycin A injection); (2) ATP production: the last rate measurement before oligomycin injection minus the minimum rate measurement after oligomycin injection; (3) maximal respiration: the maximum rate measurement after FCCP injection minus non-mitochondrial respiration rate; and (4) spare respiratory capacity: the maximum respiration minus basal respiration. All values of OCR parameters calculated were normalized to the quantified protein content.

### RNA isolation

Total RNA was extracted with the QIAzol lysis reagent (Qiagen Inc., Valencia, CA) as described previously^81^. The quantity and purity of RNA were validated using a Nanodrop spectrophotometer (Thermo Fisher Scientific). All RNA samples used were treated with DNase (Thermo Fisher Scientific) to remove potential DNA contamination. The extracted RNA was used for both the microarray and reverse transcription-quantitative polymerase chain reaction (RT-qPCR) assays as described below.

### Microarray assay

A human message RNA (mRNA) Expression Microarray assay (V4.0) and data analysis services were provided by Arraystar Inc. (Rockville, MD) to assess and compare the global abundance of 17,195 mRNAs across iPSC-derived 2-month cerebral organoids treated with and without ethanol. Before performing the microarray assay, RNA underwent quality control analysis of RNA integrity, quantity, and purity, in addition to the detection of genomic DNA contamination and the efficacy of probe labeling. The RNA was converted to cDNA, synthesized from RNA via reverse transcription, and hybridized to Arraystar’s custom mRNA probes. The microarrays used transcript-specific, single probe per transcript design and Agilent SurePrint manufacturing technology. The microarrays were scanned by Agilent Scanner G2505C. Agilent Feature Extraction software (version 11.0.1.1) was used to analyze the acquired array images; with built-in multiple metrics for quality control flags including whether a feature is positive and significant above background, uniform, saturated, population outlier, background uniformity, and background population outliers. The microarray raw intensities were normalized by quantile normalization method as previously described^82^. The log2 transformed, normalized intensities were used for differential analysis. Log2 transformed normalized intensities fit normal distribution for *t*-test better than intensities in linear scale. P-values were calculated using an unpaired *t*-test. *T*-test performs reasonably well for clearly differentially expressed and better-expressed genes for top differential expression gene candidate selection. Criteria for detailed statistical analysis for the mRNA microarray quality control are available through Arraystar (www.arraystar.com). Fold change was calculated using the absolute ratio of the normalized intensities between two conditions. Differentially expressed mRNAs were designated by expressing above ±2.0 fold change and *P* < 0.05 between control and ethanol-treated cerebral organoids were depicted in heatmaps. False discovery rate (FDR) was calculated by adjusting p-values to account for multiple testing of many genes using Benjamini-Hochberg Procedure. Gene abundance from the array assay was confirmed by RT-qPCR.

### Bioinformatic analysis of differentially expressed mRNAs and their associated signaling pathways

Ingenuity Pathway Analysis (IPA) was performed similarly to previous publications^40,63^. To propose the potential connection between ethanol-induced altered gene expression and pathways relevant to neurodevelopment and neurodegeneration, mRNAs abundance profiles from each group underwent bioinformatics assessment using the IPA software (Qiagen Bioinformatics). From the current dataset of raw gene abundance values for 17,195 mRNAs, only mRNAs with a ±2.0 fold change difference (*P* < 0.05) between groups were input into the IPA software. All ethanol-induced dysregulated gene-related disease and biology function pathways (with Fisher’s exact test *P* < 0.05) were displayed and analyzed. To more closely focus on specific molecular signaling networks in neurodevelopment and central nervous disorders, the abnormally expressed genes and their potential regulation relationship were drawn using the IPA software based on previously reported upstream regulators, downstream effects, and a respective gene’s involvement in established pathways based on the literature and databases. All dysregulated gene profiles were included in Supplemental Table 1. The phenotypic relevance of ethanol-induced abnormally expressed genes to neurodevelopment, nervous system physiology, and neurodegeneration was determined by literature searches through the PubMed database and summarized in Supplemental Table 2.

### Reverse transcription-quantitative polymerase chain reaction (RT-qPCR)

Complementary DNA (cDNA) was synthesized from the isolated RNA by the RT^2^ First Strand Kit (QIAGEN, Hilden, Germany). For the PCR assay, cDNA was mixed with QuantiTect SYBR (a green fluorescent cyanine dye that has a high affinity for double-stranded DNA) Green PCR Master Mix (QIAGEN), primers, and RNase-Free Water (QIAGEN). PCR was performed on the iCycler instrument (Bio-Rad, Hercules, CA, USA) for 10 min at 95 °C followed by 40 cycles (95 °C for 20 s, 60 °C for 30 s, and 72 °C for 30 s). All PCR reactions were performed in triplicate. The mean cycle threshold (Ct) values of triplicate wells for each sample were collected and the expression data were normalized to the endogenous control 5S rRNA and assessed using the comparative 2^−ΔΔCt^ method. All primers were purchased from Thermo Fisher Scientific and the sequences of the primers are listed in the table below.

Primer sequences for RT-qPCR

**Table:** 

Gene	Forward primer sequence(5′ to 3′)	Reverse primer sequence (5′ to 3′)	PCR product length (bp)
*EN2*	CGCGCAGCCCATGCTCTGGC	GCTTGTCCTCTTTGTTCGGGTTC	116
*GABRG3*	ATGCTACGCCAGCAAGAACAGC	GGCGAAGACAAACAGGAAGCAC	149
*DPYSL2*	ACTGCCCAGAAGGCTGTAGGAA	CAGCCACAAACTGGTTCTCATCC	135
*KCNB2*	GGAGAAACCTAACTCATCAGTGG	CCAAATTCGTCCGTTTCCTGCAG	125
*RIMKLA*	GTCATAGGCTCTATGCTTCGCTG	GGACACCTGAATAGCCAACTGC	123
*5s*	CGCCCGATCTCGTCTGAT	AAAGCCTACAGCACCCGGTA	96

*EN2* engrailed homeobox 2, *GABRG3* gamma-aminobutyric acid type A receptor gamma3 subunit, *DPYSL2* dihydropyrimidinase like 2, *KCNB2* potassium voltage-gated channel subfamily B member 2, *RIMKLA* ribosomal modification protein rimK like family member A.

### Statistical analysis

Many in vitro cell culture studies used 3–4 samples per group^51,83–86^. Previous studies showed that ethanol significantly induced neurotoxicity, such as neuronal death, based on the data that were obtained from *n* = 3^87,88^. Following similar studies that use *n* = 4 to see differences, our study used *n* = 4 unless stated otherwise. All data are presented as mean ± standard error of the mean. Statistical analysis was conducted using GraphPad Prism version 7.0. Statistical difference was analyzed by a student’s unpaired two-tailed *t*-test when comparing two groups. *P* < 0.05 was considered statistically significant for all tests. Statistical outliers were determined by the Robust regression and outlier removal method. All data presented are normally distributed based on either D’Agostino or Pearson or Shapiro-Wilk normality test. Variation within each group was not significant.

## Results

### iPSC-derived 3D cerebral organoids develop over time and are composed of different brain cell types

iPSCs grew as colonies in normal stem cell culture conditions and expressed pluripotent cell markers OCT4 and SSEA4 (Fig. 1A). Over the 2-month differentiation, singularized iPSCs sequentially developed to embryoid bodies, to neuroepithelial tissue, to cerebral organoids. This development was accompanied by an increase in tissue size (Fig. 1B), and an emergence of neuroepithelial stem cells and neurons. Specifically, at 1 month, immunofluorescence staining images indicate that cerebral organoids were primarily composed of PAX6-positive neuroepithelial stem cells, relative to MAP2-positive neurons. At two months, PAX6-positive neural stem cells decreased and MAP2-positive neurons increased (Fig. 1C). In 2-month cerebral organoids, synapses, indicated by punctate distribution signals of presynaptic maker synapsin I, were visible between the networks of MAP2-positive neurons (Fig. 1D-a). The neurons were immature as evidenced by the expression of doublecortin (Fig.1D-b). Doublecortin is a microtubule-associated protein expressed in immature neurons and turned off before neurons reach maturity^89,90^. S100β-positive astrocytes (Fig. 1D-c) were also seen in 2-month-old cerebral organoids throughout the tissue.

### iPSC-derived 3D cerebral organoids mimic human fetal brain development and contain organized tissue

Cerebral organoids could recapitulate human brain development at a considerable level of detail in many aspects such as the establishment of discrete regions (e.g., forebrain and choroid plexus) of the central nervous system. Cerebral organoids were most similar to layers of neurons called the cortex and choroid plexus. Our following data showed the consistent results with previous reports on iPSC-derived cerebral organoids^62,65,91,92^. The 2-month cerebral organoids exhibit well-organized elaborate cellular laminar organization and architectures, i.e., neural stem cells (NSCs) and neurons were located in the different layers of the organoid tissues. SOX2-positive NSCs were located on the apical side and differentiated MAP2-positive neurons from NSCs were located on the basal side (Fig. 1E-a). Neuronal cells did also express the neuronal markers CTIP2 and FOXP2 (Figs. 1E-b and 1E-c, respectively). CTIP2 identifies migrating neurons and deep cortical layers V/VI, whereas FOXP2 expression in the developing cortex is restricted to subpopulations of post-mitotic neurons in deep cortical layers (V/VI)^73,93^. These elaborate cellular laminar organization and architectures are very similar to what was observed in human brains^94–96^. Moreover, the organoids contained forebrain- and choroid plexus-like brain regions (Fig. 1F-a and b, respectively).

### Ethanol induces apoptosis and ultrastructure changes on cerebral organoids

Two-month iPSC-derived cerebral organoids were treated with increasing concentrations of ethanol. Since we sealed our culture dish with parafilm and freshly prepared ethanol mixture each time, alcohol maintained the same concentration over 24-h ethanol exposure. However, ethanol concentration in the medium was dramatically decreased when the culture dishes were not sealed (Fig. 2A). Western blot assays showed that ethanol exposure for 6 h significantly induced the increased cleaved caspase 3, an apoptosis indicator, protein expression in 2-month cerebral organoids in a dose-depend manner. Apoptosis induction in the organoids started at an ethanol dose of 115 mg/dL, and higher doses of ethanol increased the apoptosis rates (*P* < 0.05; Fig. 2B-a). The confocal images of the immunofluorescence stained tissue sections displayed more cleaved caspase 3-positive apoptotic cells in the organoids compared with the control group (Fig. 2B-b). Ethanol-induced apoptosis was consistently observed in cerebral organoids derived from two iPSC lines (Fig. 2C and Fig. 2B). Electron microscopy images revealed ethanol (230 mg/dL, 6 h)-induced ultrastructural changes of cells in the organoids (Fig. 2D). In non-ethanol treated control organoids the microfibrils appeared plentiful and well organized within the cells. Mitochondria with intact cristae and dense matrix were plentiful and the synapse structure appeared without any obvious pathological changes. In contrast to this and in agreement with the Western blot and immunofluorescence staining assays, the electron microscopy images of the ethanol-treated organoids show numerous shrunken, apoptotic cells with densely staining nuclei showing condensed and fragmented chromatin. The formation of classic apoptotic ‘half-moon’ nuclear morphology bodies was also seen in some cells. The mitochondria cristae were often disrupted, and the density of the mitochondrial matrix was decreased. There was abundant glycogen compared with the control cells. Microfibrils appeared disorganized or not visible. The degenerating synaptic terminals with cytosolic disruption were filled with electron-dense aggregates (Fig. 2D). These data suggest that ethanol exposure resulted in pathological changes of cerebral organoids including cell apoptosis, decreased mitochondrial morphology and matrix density, disorganized microfibrils and synapse degeneration.

### Ethanol induces apoptosis in neurons but not in astrocytes on cerebral organoids

In order to identify vulnerability of different brain cells in the organoids to ethanol exposure (230 mg/mL, 6 h), the organoid sections were subjected to immunofluorescence and TUNEL staining for visualizing apoptotic cells, neurons and astrocytes. Ethanol treatment induced an increase of TUNEL-positive apoptotic cells, which is consistent with the activated caspase 3 data (Figs. 2B and C). In some cells, TUNEL–positive apoptotic and NeuN-positive neuron signals were co-localized (Fig. 3A). We also found that some TUNEL-positive cells did not express NeuN indicating that neurons may lose NeuN signals at a later stage of apoptosis. We did not find the increased TUNEL and S100β double-positive apoptotic astrocytes in the ethanol-treated cerebral organoids compared with control organoid tissues (Fig. 3B). These data suggest that neurons are more vulnerable to ethanol treatment in the aspect of the apoptotic response.

### Ethanol alters mitochondrial oxidation of cells in cerebral organoids

We investigated the effect of ethanol (230 mg/mL, 6 h), on mitochondrial respiration function by the sequential addition of oligomycin, FCCP, and antimycin A (Figs. 4A and B). We observed that ethanol significantly decreased 2-month cerebral organoids’ OCR values that were linked to basal respiration, ATP production, maximal respiration, and spare respiratory capacity (*P* < 0.05), suggesting abnormal mitochondrial oxidative capacity (Fig. 4C). We also found that non-mitochondrial respiration (Fig. 4C) was increased following ethanol exposure.

### Ethanol induces an alteration of mRNA profiles in cerebral organoids

To dissect the potential molecular mechanisms of ethanol-induced neurotoxicity in the organoids, we performed a microarray assay and analyzed the expression of 17,195 mRNAs. The integrity of total RNAs isolated from 4 control- or 4 ethanol (230 mg/mL, 6 h)-treated 2-month cerebral organoids were analyzed using denaturing agarose gel electrophoresis. The gel image exhibits three RNA bands of 28s, 18s, and 5s, indicating no degradation of the RNAs used for array assay (Supplemental Fig. 1A). The box plots display similar distributions of normalized RNA intensity values across eight control and ethanol samples, suggesting that the microarray data can be used for further analysis (Supplemental Fig. 1B). Among 17,195 mRNAs analyzed, there were 199 dysregulated mRNAs (162 upregulated and 37 downregulated) in the ethanol-treated organoids (fold change > ±2, *P* < 0.05) (Supplemental Table 1). The heatmap displays the relative expression patterns of these differentially expressed in all control and ethanol-treated cerebral organoid samples (Fig. 5A). The expression level of 5 randomly selectively altered mRNAs [GABRA2 (gamma-aminobutyric acid type A receptor alpha2 subunit), GABRG3 (gamma-aminobutyric acid type A receptor gamma3 subunit), KCNB2 (potassium voltage-gated channel subfamily B member 2), RIMKLA (ribosomal modification protein rimK like family member A), and VSNL1 (visinin like 1)] was further validated using RT-qPCR (*P* < 0.05; Fig. 5B), and the data were consistent with what was obtained from the microarray assay.

### Potential signaling pathways for ethanol-induced dysregulated mRNA involvement

Bioinformatics analysis of ethanol-induced altered gene-related signaling in 2-month cerebral organoids was conducted using the IPA-Diseases and Functions Analysis. This assay was used to predict the effected disease and biology function based on dysregulated gene expression conferred by ethanol. IPA shows that the ethanol-induced dysregulated miRNAs were related to the following 37 diseases and biology function signaling pathways which are associated neurological functions and diseases, tissue injury, development, and basic physiological processes: Organismal Injury and Abnormalities; Cell Morphology; Cellular Development; Cellular Growth and Proliferation; Nervous System Development and Function; Organismal Development; Tissue Development; Neurological Disease; Tissue Morphology; Cellular Assembly and Organization; Cellular Function and Maintenance; Developmental Disorder; Cell-To-Cell Signaling and Interaction; Hereditary Disorder; Psychological Disorders; Organ Morphology; Organismal Survival; Cellular Compromise; Post-Translational Modification; Protein Degradation; Protein Synthesis; Behavior, Cellular Movement, Cell Death and Survival, Embryonic Development; Organ Development; Organismal Functions; Gene Expression; Molecular Transport; Protein Trafficking; Cell Signaling; Cell Cycle; DNA Replication; Recombination, and Repair; Lipid Metabolism; Small Molecule Biochemistry; Metabolic Disease; and Energy Production (Fig. 6A-a). All abnormally expressed genes that are related to 37 diseases and biological function signaling pathways above were included in Supplemental Table 3. The following six representative disease and biological function, and their related dysregulated genes were depicted in Fig. 6A, b: (1) cell development (e.g., cellular proliferation, neuron and synapse development, axonogenesis, and differentiation of nervous system), (2) nervous development and function (e.g., brain morphology, migration of neurons, branching and morphology of axons, and long term synaptic depression), (3) neurological diseases (e.g., cognitive dysfunction, ischemia stroke, epileptic seizure, neurological disorder, attention deficit hyperactivity disorder), (4) cell-to-cell signaling and interaction (e.g., neurotransmission, potential, and organization of synapse), (5) psychological disorders (e.g., anxiety, mental retardation, speech and language disorders, bipolar depression), (6) behavior (e.g., social learning, contextual conditioning, abnormal social behavior, mating behavior), and (7) cell death and survival (e.g., apoptosis and neurodegeneration). These diseases and biology function signaling have been reported to be related to ethanol-induced neurotoxicity^6,7,97–102^, and might participate in ethanol-induced neurotoxicity such as apoptosis, mitochondrial dysfunction, and degenerated synapse as we observed in the cerebral organoids (Figs. 2 to 4). In addition, among total 199 ethanol-induced dysregulated genes, 187 of these genes were previously reported as critical to neurodevelopment and/or implicated in neurodegeneration (Supplemental Table 2).

### Potential gene network of dysregulated genes in neurodevelopment and neurological diseases

In order to find the potential molecular mechanisms of ethanol-induced brain injury, we performed an IPA network analysis and found 13 signaling networks ranked by the numbers of dysregulated genes. The following top three signaling networks include 26, 23, and 22 ethanol-induced altered genes, respectively: (1) Developmental Disorder, Hereditary Disorder, Neurological Disease network (Fig. 6B-a and b), (2) Cell Morphology, Cell-To-Cell Signaling and Interaction, Nervous System Development and Function (Fig. 6B-c), and (3) Cell Morphology, Cell Signaling, Cellular Assembly and Organization (Fig. 6B-d). The gene-gene interaction networks in the signaling pathways above were displayed in Fig. 6.

## Discussion

This study is pioneering to extend animal model studies on FASDs to human models of iPSC-derived cerebral organoids. Specifically, we quantified the downstream toxic effects of a binge drinking-like ethanol exposure on neural pathology phenotypes and signaling pathways. We found that ethanol induced the following neurotoxicity on the 2-month cerebral organoids at tissue, cellular, subcellular, bioenergetic metabolism, and gene expression levels. Ethanol exposure for 6 h resulted in apoptosis in the organoids in a dose-dependent manner. Neurons were more vulnerable to ethanol-induced cell death than astrocytes. Ethanol exposure also resulted in ultrastructural changes, mitochondrial dysfunction, and abnormal genome-wide gene profiles. Bioinformatics analyses suggest that ethanol-induced dysregulated genes are associated with not only cell apoptosis, but also other neurodevelopment and neurodegeneration signaling. Extensive regulative networks of these genes might be involved in ethanol-induced brain injuries such as abnormal brain development, cognition, behavior, and psychology. The findings in this study provide a better understanding of the neurotoxic effects of binge drinking during pregnancy, specifically on mitochondrial dysfunction and extensive signaling networks of the developing brain.

Since 2013 when Dr. Knoblich’s lab developed an in vitro system of human cerebral organoids from iPSCs, we and many other researchers have conducted additional characterization of this model, showing that cerebral organoids were similar to developing human brains in the aspects of complexity, structure, and function furthering the study of neurodevelopment and neurological diseases^40,51,55,58,59^. The generation of cerebral organoid is a highly dynamic model of the embryonic developing brain over time^103^. Notably, cerebral organoids closely resemble neurons in the cortex and choroid plexus^36,70,104^. Our organoids exhibited similar developmental patterns, contained multiple brain cell types (e.g., NSCs, neurons, and astrocytes), and showed distinct multi-layered, cortical-like neuronal zone and choroid plexus (Fig. 1) as previously reported^91,105,106^. As neurodevelopment is difficult to study in vivo, the use of human iPSC-derived cerebral organoids for modeling brain development and related neurological diseases has offered significant breakthroughs.

Binge drinking or consuming a large amount of alcohol in a short period of time is an increasingly significant public health issue. Animal studies found that binge-like drinking patterns, in which the fetus was exposed to high BACs over relatively short periods of time, were harmful as evidenced by acute apoptosis and long-term cognitive dysfunction^107–110^. One of the main gaps in knowledge for ethanol-induced brain injury is regarding dose-response relationships. No amount of alcohol use is known to be safe for a developing baby before birth^6,111^. In this study, we exposed 2-month organoids with different concentrations of ethanol for 6 h to mimic binge drinking ethanol exposure. We found that ethanol dose-dependently induced apoptosis (Fig. 2) as observed previously in cultured fetal brain-derived cells^44^. The apoptosis was confirmed by multiple approach analyses including immunofluorescence staining, TUNEL staining, Western blot, and electron microscopy imaging (Figs. 2 and 3). The lowest ethanol concentration in the culture medium to trigger apoptosis was 110 mg/dL which is equivalent to the BACs resulted from 3 to 5 standard drinks, depending on body weight^112^, suggesting that this drinking pattern might be a risk to the fetal brains in the aspect of apoptosis.

Apoptosis is a commonly recognized side effect of ethanol in developing animals. It has been shown that in utero exposure of the fetal non-human primate macaque brain to alcohol on a single occasion triggered widespread acute apoptotic death of neurons but not astrocytes^113,114^. The neuron apoptosis was also observed in ethanol-treated developing mouse brains^115^. However, there are no reports regarding the apoptotic vulnerability of different human brain cells in response to ethanol exposure. In the current study, we had similar findings of ethanol-induced apoptosis in neurons (Fig. 3A) but not in astrocytes (Fig. 3B) as seen in animal models^113–115^. The potential mechanisms underlying selective vulnerability in neurodegeneration are complex and incompletely understood. Ethanol has both N-Methyl-D-Aspartate (NMDA) antagonistic and gamma-aminobutyric acid gamma-aminobutyric acid (GABA)-mimetic properties^115,116^. We and others showed several classes of drugs such as anesthetics propofol and ketamine, which has both NMDA antagonist and GABA agonist properties, triggered widespread neuroapoptosis throughout the developing animal brains and human stem cell-derived neurons^74,75,84,117^. Thus, it is possible that ethanol and anesthetic drugs exert their apoptogenic action by due mechanism-blockade of NMDA receptors and hyperactivation of GABA receptors. We did not observe an increased astrocyte apoptosis, but this does not rule out a possible adverse influence of ethanol on astrocyte function. Our previous cell culture study showed that propofol did not induce rat astrocyte apoptosis but decreased the secretion of growth factors and cytokines (e.g., brain-derived neurotrophic factor and vascular endothelial growth factor C)^74^. Knowing the specific cellular vulnerability will help to develop more reasonable protective strategy targeting neurons. Downregulated paracrine factors have been shown to play important roles in neuron survival, learning, and memory^118^. Whether ethanol induces such side effects on human astrocytes in organoids remains for future investigation.

In addition to understanding whether and how ethanol causes cell death, we sought to understand how ethanol disrupts the remaining living cells. We observed the ultrastructure of organoids and found the disorganization of cellular structure in cells and degenerated synapses (Fig. 2). The cytoskeleton encompasses a multitude of filamentous proteins, forming structures that impart mechanical strength, allow intracellular transport and spatial organization, connect the cell to its environment, and generate forces that permit movement^119^. The neuronal cytoskeleton, in specific, not only provides the structural backbone of neurons but also plays a fundamental role in maintaining neuronal functions. Dysregulation of neuronal architecture was evident in neurological diseases such as mental disorders and neurodegeneration^120^. These changes might result in the disruption of protein trafficking, loss of synapses, and the death of neurons, other potential pathology, and progression of ethanol-induced disturbance of brain development such as decreased dendritic spine density and cell migration shown in previous animal studies by others^121,122^.

Mitochondria occupy unique roles in regulating brain function and metabolism on various levels, such as regulating ATP production, and contributing to redox balance and calcium homeostasis. Synthesized ATP by brain cell mitochondria fuels energy-dependent intracellular reactions (such as ion transport, vesicle release, and neurotransmitter biosynthesis and uptake), contributing to intracellular signaling, synaptic process, and brain function. Most defects of mitochondria are linked to neurodegenerative and metabolic diseases^123^. Previous findings from animal studies suggest that mitochondria appear to be a major target of ethanol toxicity in the brain. Reactive oxygen species (ROS) and reactive nitrogen species (RNS)-associated disturbances of the integrity of the mitochondrial membrane and decreased mitochondrial genes were important mediators for the ethanol-induced brain cell injuries^124–126^. However, mitochondrial respiration capacity was not investigated in the FASD research fields. We found that in ethanol-treated organoids, the mitochondria cristae were often disrupted, and the density of the mitochondrial matrix decreased (Fig. 2). In addition, mitochondrial oxygen consumption rates, which are linked to basal respiration, ATP production, maximal respiration, and spare respiratory capacity (Fig. 4), were significantly reduced following ethanol exposure on cerebral organoids. Basal respiration measures the cells’ relative utilization of mitochondrial respiration under resting conditions. ATP linked respiration measures the oxygen consumption linked directly to ATP production. Maximal respiration measures the cells’ relative utilization of mitochondrial respiration when stressed. Spare respiratory capacity is used for the measurement of the cells’ ability to respond to an energetic demand and is an indicator of cell fitness or flexibility^127^. If the threshold for the basal respiration is breached then cell death occurs^128,129^. Whether cells can utilize the maximal electron transport activity for ATP synthesis will depend on the capacity of the components of the electron transport chain and oxidative phosphorylation system^130^. The decreased OCR parameters observed in cerebral organoids suggest the ethanol-induced mitochondrial dysfunction possibly due to low ATP demand, low mitochondrial mass, a lack of substrate availability, or damage to the electron transport chain, which would impede the flow of electrons and result in a lower OCR. We also found that ethanol increased non-mitochondrial respiration in organoids (Fig. 4). Non-mitochondrial OCR is an index of oxygen-consuming processes that are not mitochondria and attributed to enzymatic reactions outside of mitochondria such as cyclooxygenases, lipoxygenases, cytochrome P450s and (nicotinamide adenine dinucleotide phosphate) NADPH oxidases. This parameter increased in response to cell stressors such as ROS and RNS. An increase of non-mitochondrial OCRs is regarded as negative indicators of bioenergetic health^127,129^. There is ample evidence showing that impaired mitochondrial function is associated with neurodegeneration. Mitochondria dysfunctions such as the ones described above were also associated with different psychiatric disorders, such as bipolar disorder, depression, and schizophrenia^131^. Our data suggest that cerebral organoids undergo metabolic stress, possibly resulting in acute cell degeneration/apoptosis observed and other living brain cell function in the organoids (Figs. 2–3), contributing to long-term cognitive dysfunction and psychological disorders seen in FASDs patients.

The understanding of the pathophysiological phenotypes and downstream molecular pathways of ethanol-induced developing brain injury is limited. In order to unravel this mechanism, in this study we analyzed genome-wide gene expression and found that among 17,195 mRNAs analyzed, there were 199 genes significantly altered by ethanol. These data suggest that binge drinking-like exposure induces significantly abnormal gene expression profiles (Fig. 5). Brain development begins within the first month of gestation. During brain development, many development events progressively occur such as neural stem cell proliferation, neurogenesis, synaptogenesis, cell migration, and network connections). Environmental exposures, such as ethanol, can influence the fate of the brain development process (e.g., neural cell survival, differentiation, migration, and network connections) and result in abnormal brain function. Animal studies suggest that ethanol could target various brain cells, and induce not only cell death but also much other adverse consequence such as altered neuronal structural and functional plasticity, inhibition of neurite outgrowth, reduced neurotrophic factors and neurotrophic expression, decreased synapse plasticity, delayed myelinations, and astrocyte proliferation and differentiation^6^. Thus, the current study on the analysis of the signaling pathways based on altered mRNAs was not limited to focus on apoptosis alone but predicted pathways linked to multiple neurological outcomes.

Bioinformatics analyses disclosed the association of these dysregulated genes with 37 notable pathways such as psychological disorders (e.g., anxiety, mental retardation, speech and language disorders, and bipolar depression), nervous system development, function, and diseases, organismal injury and abnormalities, and cellular development (e.g., synapse development and differentiation of nervous system). Many ethanol-induced dysregulated genes were predicted to form regulative networks in cell function, communication, development, and neurological disease pathways (Fig. 6). Thus, in addition to apoptosis outcome, ethanol possibly interferes with many other cell activities and results in abnormal development of cerebral organoids. In addition, we found that 187 out of 199 dysregulated genes from our study were previously reported in the literature as critical to cell biology, physiology, neurodevelopment, and/or implicated in neurodegeneration (Supplemental Table 2). For instance, ADAM metallopeptidase domain 21 (ADAM21) is associated with neurogenesis and axonal growth in postnatal development^132^. Potassium voltage-gated channel subfamily C member 1 (KCNC1) mediates the voltage-dependent potassium ion permeability of excitable membranes and variants prevent neuronal inhibition, which is associated with epilepsy, ataxia, intellectual disability, and developmental delay^133^. Myocyte enhancer factor 2C (MEF2C) is highly expressed in developing cortical excitatory neurons, variants linked to autism, intellectual disability, and schizophrenia^134^. The existing information on the role of these genes in brain function, neurodevelopment, and non-ethanol neurological diseases, and predicted regulative networks in the defined signaling pathway (Fig. 6) are extremely valuable in providing new insights on potential phenotypes related to ethanol-induced toxicity in human brain cells. The severity of the FASD symptom increases over time. Currently, there is no treatment, or approved protective therapy for FASDs, there have been several trials that looked into the potential of antioxidants, and other supplements like folic acid, L-glutamine, boric acid, and choline^135^. Understanding the ethanol-induced pathological changes and underlying the mechanisms using cerebral organoids will provide valuable insight into the development of reasonable prevention and intervention strategies.

Collectively, we show that human iPSC-derived 3D cerebral organoids can recapitulate complex features of the brain and offer an unprecedented opportunity to model ethanol-induced complex human brain neurotoxicity that affects brain development and multiple cell types, and the underlying molecular and cellular mechanisms. This current study is one of the first to extend animal model studies on FASDs to the human model of cerebral organoids at the tissue, cellular, subcellular, and gene levels. We for the first time revealed that human brain cells exhibit the apoptosis response to ethanol in a dose- and brain cell type-dependent manner, mitochondrial dysfunction, ultrastructure changes of cells such as degenerated synapse. The pathway analyses of ethanol-induced abnormal gene expression profiles improve our molecular mechanistic understanding of various pathophysiological processes following binge drinking ethanol exposure. This study may also contribute to the development of intervention and prevention strategies to reduce or eliminate the harmful effect of alcohol exposure.

## Supplementary information

Supplemental Materials and Methods

Supplemental Figure 1

Supplemental Table 1

Supplemental Table 2

Supplemental Table 3
