# Correction to: Modeling alcohol-induced neurotoxicity using human induced pluripotent stem cell-derived three-dimensional cerebral organoids

**DOI:** 10.1038/s41398-021-01214-z

**Published:** 2021-02-01

**Authors:** Thiago Arzua, Yasheng Yan, Congshan Jiang, Sarah Logan, Reilly L. Allison, Clive Wells, Suresh N. Kumar, Richard Schäfer, Xiaowen Bai

**Affiliations:** 1 Department of Cell Biology, Neurobiology & Anatomy, Medical College of Wisconsin, Milwaukee, 53226 WI USA; 2 Department of Physiology, Medical College of Wisconsin, Milwaukee, 53226 WI USA; 3 Department of Anesthesiology, Medical College of Wisconsin, Milwaukee, 53226 WI USA; 4 Department of Microbiology, Medical College of Wisconsin, Milwaukee, 53226 WI USA; 5 Department of Pathology, Children’s Research Institute Imaging Core, Neuroscience Imaging Facility, Medical College of Wisconsin, Milwaukee, 53226 WI USA; 6 Institute for Transfusion Medicine and Immunohaematology, German Red Cross Blood Donor Service Baden-Württemberg-Hessen gGmbH, Goethe University Hospital, 60438 Frankfurt am Main, Germany

Correction to: *Translational Psychiatry*

10.1038/s41398-020-01029-4 published online 13 October 2020

The original version of this article unfortunately contained several typos in the unit of ethanol concentration. The unit of ethanol concentration should be “mg/dL” but not “mg/mL”. The original article has been corrected.

