# Supplementary material for: Modeling alcohol-induced neurotoxicity using human induced pluripotent stem cell-derived three-dimensional cerebral organoids
Source: Transl Psychiatry. 2020 Oct 13;10:347. doi: 10.1038/s41398-020-01029-4 (PMC7553959; doi:10.1038/s41398-020-01029-4)
Supplement: Supplementary file 1 — Supplemental Materials and Methods [file 41398_2020_1029_MOESM1_ESM.pdf]

Supplemental Materials and Methods

3D cerebral organoid generation from iPSCs

The protocol we used for the generation of cerebral organoids was developed by Lancaster et al. [1]. Briefly, we generated organoids from iPSCs by use of chemically defined medium and plating strategies (Fig. 1) as we and other described previously [1-3]. Briefly, during the passaging process, 12,000 singularized iPSCs were re-suspended in mTeSR1 medium (150 µl cell suspension) and added to each well of the 96-well ultra-low attachment U-bottom plate. Embryoid bodies (EBs) formed in the ultra-low attachment plates for 6 days, maintained in normoxic conditions (21% O_2) at 37°C, and mTeSR1 was added every other day. On day 6, EBs were transferred to 24-well low attachment plates, and cells were cultured in neuroepithelial induction media (DMEM/F12, 1% N2 Supplement, 1% glutamine, 1% nonessential amino acids (Thermo Fisher Scientific) and 1 µg/ml Heparin 50 (Sigma-Aldrich, St. Louis, MO, USA) for 5 days. On day 11, neuroepithelial tissues were embedded in Matrigel™ droplets and plated on 100 mm plates in 12 ml cerebral organoid differentiation media (DMEM/F12, Neurobasal media, 0.5% N2 Supplement, 1% Glutamine, 0.5% nonessential amino acids, 1% penicillin/streptomycin, 1% B27 without vitamin A (Thermo Fisher Scientific), 0.025% insulin (Sigma-Aldrich), and 0.035% mercaptoethanol (Sigma-Aldrich) for 5 days. Plates were transferred to a spinner on day 16, and cultured long-term in 12 ml cerebral organoid differentiation media supplemented with vitamin A (DMEM/F12, Neurobasal media, 0.5% N2 Supplement, 1% glutamine, 0.5% nonessential amino acids, 1% penicillin/streptomycin, 1% B27 with vitamin A (Thermo Fisher Scientific), 0.025% insulin, and 0.035% mercaptoethanol (Sigma-Aldrich). Cerebral organoids were cultured up until 2 months, with a subset of iPSCs and 1-month-old cerebral organoids collected for imaging. iPSC colonies, embryoid bodies, neuroepithelial tissues, and cerebral organoids were examined daily under the microscope and imaged under bright field by an EVOS FL Auto microscope (Thermo Fisher Scientific).

Immunostaining analysis of iPSC, neural progenitor cells, brain cell-specific marker expression, and apoptosis on cerebral orgnaoids

iPSC cultured on the Matrigel™-coated coverslips were sequentially fixed with 4% paraformaldehyde (Electron Microscopy Sciences, Hatfield, PA, USA) for 15 min at room temperature, permeabilized with 0.5% Triton X-100 (Sigma-Aldrich) in phosphate-buffered saline (PBS) (Thermo Fisher Scientific) for 15 min, and blocked with 10% donkey serum (Millipore, Billerica, MC, USA) for 20 min at room temperature as described previously [4]. The cells were then stained with the following primary antibodies at 4 °C overnight: mouse anti-stage-specific embryonic antigen 4 (SSEA4: a pluripotent stem cell marker, Abcam ab16287, Cambridge, MA, USA and rabbit anti-octamer-binding transcription factor 4 (OCT4: a pluripotent stem cell marker, Abcam ab18976). After washing with PBS twice, the cells were incubated in the dark for one hour at 37 °C with a secondary antibody; Alexa Fluor 594 goat anti-rabbit immunoglobulin G (IgG) (Thermo Fisher Scientific) and Alexa Fluor 594 goat anti-mouse IgG (Thermo Fisher Scientific). Hoechst 33342 (Thermo Fisher Scientific) was used to stain cell nuclei. The coverslips were placed on glass slides with mounting medium (Thermo Fisher Scientific) and sealed with nail polish at the conclusion of immunostaining. Samples were imaged by laser-scanning confocal microscopy (Nikon Eclipse TE2000-U, Nikon, Minato, Tokyo, Japan).

Cerebral organoids were fixed in 10% zinc formalin 100 (Sigma-Aldrich), embedded in paraffin, and slices of 4 μm thickness were attached to glass slides. Tissues were deparaffinized by sequential washing in xylenes, hydrated in graded ethanol, and incubated in antigen retrieval solution (Dako, Glostrup Denmark) at 95°C. Following 0.5% Triton X-100-PBS washing, 10% donkey serum was used as a blocker, and the following primary antibodies were used: paired box protein 6 (PAX6, a neural stem cell marker [5, 6]; rabbit anti, Biolegend 901301, San Diego, CA, USA), microtubule-associated protein 2 (MAP2, a neuron-specific marker; mouse anti, Abcam ab11267), synapsin1 (a synapse marker; rabbit anti, Cell signaling #5297, Danvers, MA, USA), S100 calcium-binding protein β (S100β, an astrocyte marker; rabbit anti, Abcam ab52642), doublecortin (an immature and migrating neuron marker [7, 8]; rabbit anti, Abcam ab18723), SRY-Box Transcription Factor 2 (SOX2, a neural stem cell marker

[9]; mouse anti, Sigma MAB4343), SOX2 (rabbit anti, abcam ab97959), Forkhead box G1 (FOXG1, a forebrain marker [6, 10-12]; rabbit anti, Abcam ab18259), prealbumin (also called TTR, a choroid plexus marker [6, 13]; sheep anti, Abcam ab9015), COUP-TF-interacting protein 2 (CTIP2, a V/VI cortical layer migrating neuron marker [6, 11, 12]; rabbit anti, Atlas Antibodies HPA049117, Bromma, Sweden), and FOXP2 (a VI cortical layer neurons marker [14, 15]; goat anti, Thermo Fisher Scientific PA5-18615), and cleaved (or activated) caspase 3 (an apoptosis marker; rabbit anti, Cell Signaling 9664L). The corresponding secondary antibodies were Alexa Fluor-488 or Alexa Fluor-594 nanometer conjugated IgG (Thermo Fisher Scientific). Hoechst 33342 (Thermo Fisher Scientific) was used to stain cell nuclei. Coverslips and mounting medium (Thermo Fisher Scientific) were placed over the tissues and sealed with nail polish at the conclusion of immunostaining. Slides were imaged by Olympus slide scanners (Olympus, Shinjuku City, Tokyo, Japan).

Immunostaining and terminal deoxynucleotidyl transferase-mediated deoxyuridine triphosphate in situ nick end labeling (TUNEL) analysis of vulnerability of neurons and astrocytes to ethanol treatemnt

Cerebral organoid tissue sections were deparaffinized and incubated in antigen retrieval solution, and blocked with 10% donkey serum as described above. In order to identify which types of brain cells undergo apoptosis following ethanol exposure, the sections received TUNEL staining following immunofluorescence staining procedure. The primary antibodies [neuronal nuclear antigen (NeuN, a neuron marker; mouse anti, Millipore MAB377, Billerica, MA, US) or S100β (an astrocyte marker; rabbit anti, Abcam ab52642), and the corresponding secondary antibodies (Alexa Fluor 488-conjugated mouse or rabbit IgG (Thermo Fisher Scientific) were used in immunofluorescence staining. To assess cell death in the organoids, an in situ Cell Death kit, TMR red (Roche Applied Bio Sciences, Indianapolis, IN) was used following instructions provided by the manufacturer as we previously described [16, 17]. This kit utilizes a process known as TUNEL to label the free 3′-OH termini of single and double stranded DNA breaks. The polymerase, terminal deoxynucleotidyl transferase (TdT) adds modified nucleotides to the free 3′-OH termini which are secondarily labeled with a fluorescent

marker. This allows for the visualization of TUNEL-positive apoptotic cells on a fluorescent microscope. The organoid sections were stained for 30 minutes with Hoechst 33342 to label the cell nuclei. Coverslips and mounting medium were placed over the tissues and sealed with nail polish at the conclusion of immunostaining. Slides were imaged by Olympus slide scanners.

References

1. Lancaster MA, Knoblich JA. Generation of cerebral organoids from human pluripotent stem cells. Nat Protoc. 2014; 9(10)**:** 2329-40.
2. Logan S, Arzua T, Yan Y, Jiang C, Liu X, Yan L *et al.* Dynamic characterization of structural, molecular, and electrophysiological phenotypes of human-induced pluripotent stem cell-derived cerebral organoids, and comparison with fetal and adult gene profiles. Cells. 2020; 9, 1301.
3. Logan S, Arzua T, Yan Y, Jiang C, Liu X, Yu LK *et al.* Dynamic Characterization of Structural, Molecular, and Electrophysiological Phenotypes of Human-Induced Pluripotent Stem Cell-Derived Cerebral Organoids, and Comparison with Fetal and Adult Gene Profiles. Cells. 2020; 9(5).
4. Kikuchi C, Bienengraeber M, Canfield S, Koopmeiner A, Schafer R, Bosnjak ZJ *et al.* Comparison of cardiomyocyte differentiation potential between type 1 diabetic donor- and nondiabetic donor-derived induced pluripotent stem cells. Cell Transplant. 2015; 24(12)**:** 2491-504.
5. Osumi N, Shinohara H, Numayama-Tsuruta K, Maekawa M. Concise review: Pax6 transcription factor contributes to both embryonic and adult neurogenesis as a multifunctional regulator. Stem Cells. 2008; 26(7)**:** 1663-72.
6. Renner M, Lancaster MA, Bian S, Choi H, Ku T, Peer A *et al.* Self-organized developmental patterning and differentiation in cerebral organoids. EMBO J. 2017; 36(10)**:** 1316-29.
7. Ayanlaja AA, Xiong Y, Gao Y, Ji G, Tang C, Abdikani Abdullah Z *et al.* Distinct Features of Doublecortin as a Marker of Neuronal Migration and Its Implications in Cancer Cell Mobility. Front Mol Neurosci. 2017; 10**:** 199.
8. Vukovic J, Borlikova GG, Ruitenberg MJ, Robinson GJ, Sullivan RK, Walker TL *et al.* Immature doublecortin-positive hippocampal neurons are important for learning but not for remembering. J Neurosci. 2013; 33(15)**:** 6603-13.
9. Ellis P, Fagan BM, Magness ST, Hutton S, Taranova O, Hayashi S *et al.* SOX2, a persistent marker for multipotential neural stem cells derived from embryonic stem cells, the embryo or the adult. Dev Neurosci. 2004; 26(2-4)**:** 148-65.
10. Mariani J, Coppola G, Zhang P, Abyzov A, Provini L, Tomasini L *et al.* FOXG1-Dependent Dysregulation of GABA/Glutamate Neuron Differentiation in Autism Spectrum Disorders. Cell. 2015; 162(2)**:** 375-90.
11. Watanabe M, Buth JE, Vishlaghi N, de la Torre-Ubieta L, Taxidis J, Khakh BS *et al.* Self-Organized Cerebral Organoids with Human-Specific Features Predict Effective Drugs to Combat Zika Virus Infection. Cell Rep. 2017; 21(2)**:** 517-32.

12. Qian X, Su Y, Adam CD, Deutschmann AU, Pather SR, Goldberg EM *et al.* Sliced Human Cortical Organoids for Modeling Distinct Cortical Layer Formation. Cell Stem Cell. 2020; 26(5)**:** 766-81 e9.
13. Aleshire SL, Bradley CA, Richardson LD, Parl FF. Localization of human prealbumin in choroid plexus epithelium. J Histochem Cytochem. 1983; 31(5)**:** 608-12.
14. Tsui D, Vessey JP, Tomita H, Kaplan DR, Miller FD. FoxP2 regulates neurogenesis during embryonic cortical development. J Neurosci. 2013; 33(1)**:** 244-58.
15. Co M, Hickey SL, Kulkarni A, Harper M, Konopka G. Cortical Foxp2 Supports Behavioral Flexibility and Developmental Dopamine D1 Receptor Expression. Cereb Cortex. 2020; 30(3)**:** 1855-70.
16. Twaroski DM, Yan Y, Olson JM, Bosnjak ZJ, Bai X. Down-regulation of microRNA-21 is involved in the propofol-induced neurotoxicity observed in human stem cell-derived neurons. Anesthesiology. 2014; 121(4)**:** 786-800.
17. Twaroski DM, Yan Y, Zaja I, Clark E, Bosnjak ZJ, Bai X. Altered Mitochondrial Dynamics Contributes to Propofol-induced Cell Death in Human Stem Cell-derived Neurons. Anesthesiology. 2015; 123(5)**:** 1067-83.
