# Supplementary material for: Modeling alcohol-induced neurotoxicity using human induced pluripotent stem cell-derived three-dimensional cerebral organoids
Source: Transl Psychiatry. 2020 Oct 13;10:347. doi: 10.1038/s41398-020-01029-4 (PMC7553959; doi:10.1038/s41398-020-01029-4)
Supplement: Supplementary file 2 — Supplemental Figure 1 [file 41398_2020_1029_MOESM2_ESM.pdf]

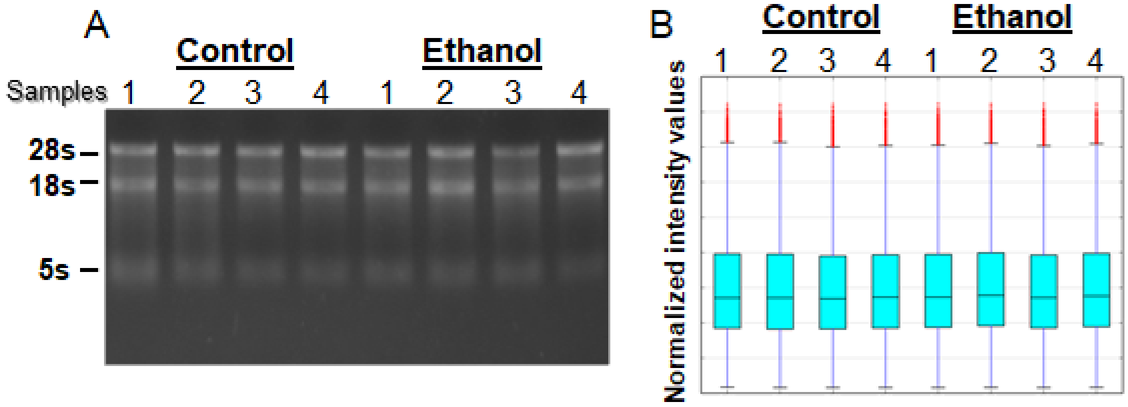

Supplemental Figure 1. Microarray analysis of 17,195 messenger RNA (mRNA) expression in the cerebral organoids. (**A**) The images of denaturing agarose gel electrophoresis of total RNAs isolated from 4 control- or 4 ethanol-treated 2-month cerebral organoids. The 3 ribonucleic acid (RNA) bands (28s, 18s, and 5s) were prominent, indicating no degradation of the RNA used for array assay. (**B**) The box plots display similar distributions of normalized RNA intensity values across all control and ethanol samples, suggesting that the microarray data can be used for further analysis. Each sample box plot includes a box with a central line and two tails. The central line refers to the median of RNA intensity values and the tails represent the upper and lower quartiles.
