# Supplementary material for: Modeling alcohol-induced neurotoxicity using human induced pluripotent stem cell-derived three-dimensional cerebral organoids
Source: Transl Psychiatry. 2020 Oct 13;10:347. doi: 10.1038/s41398-020-01029-4 (PMC7553959; doi:10.1038/s41398-020-01029-4)
Supplement: Supplementary file 3 — Supplemental Table 1 [file 41398_2020_1029_MOESM3_ESM.pdf]

Supplemental table 1. Ethanol-induced dysregulated genes

GeneSymbol	Gene description	P-value	FDR	Fold Change	Regulation
ZKSCAN2	zinc finger with KRAB and SCAN domains 2	0.01	0.25	2.22	up
MAFA	MAF bZIP transcription factor A	0.04	0.29	2.20	up
ADAM21	ADAM metallopeptidase domain 21	0.01	0.25	2.02	up
TBX1	T-box 1	0.03	0.28	2.41	up
SASS6	SAS-6 centriolar assembly protein	0.01	0.25	2.01	up
DDN	dendrin	0.05	0.31	2.58	up
PDGFC	platelet derived growth factor C	0.05	0.31	2.01	up
KCNC1	potassium voltage-gated channel subfamily C member 1	0.02	0.26	2.35	up
WNT7A	Wnt family member 7A	0.03	0.29	2.15	up
LRRC8B	leucine rich repeat containing 8 VRAC subunit B	0.02	0.26	2.07	up
MARCH11	membrane associated ring-CH-type finger 11	0.01	0.25	2.47	up
C11orf87	chromosome 11 open reading frame 87	0.01	0.25	2.38	up
MEF2C	myocyte enhancer factor 2C	0.01	0.25	2.81	up
TLE1	transducin like enhancer of split 1	0.00	0.25	2.44	up
ZNF350	zinc finger protein 350	0.05	0.31	2.57	up
HSD11B2	hydroxysteroid 11-beta dehydrogenase 2	0.00	0.24	2.05	up
TMEM196	transmembrane protein 196	0.01	0.25	2.40	up
TUSC1	tumor suppressor candidate 1	0.02	0.26	3.28	up
KIRREL3	kirre like nephrin family adhesion molecule 3	0.05	0.31	2.07	up
NFS1	NFS1, cysteine desulfurase	0.04	0.30	2.23	up
ANKRD33B	ankyrin repeat domain 33B	0.03	0.27	2.50	up
SHPRH	SNF2 histone linker PHD RING helicase	0.01	0.25	2.33	up
RGPD1	RANBP2-like and GRIP domain containing 1	0.01	0.25	2.15	up
ZNF770	zinc finger protein 770	0.02	0.26	2.00	up
SLC3A1	solute carrier family 3 member 1	0.00	0.24	2.34	up
TRMT9B	tRNA methyltransferase 9B (putative)	0.04	0.30	2.50	up
PROCA1	protein interacting with cyclin A1	0.04	0.29	2.04	up
MAS1	MAS1 proto-oncogene, G protein-coupled receptor	0.01	0.25	2.23	up
AKAIN1	A-kinase anchor inhibitor 1	0.02	0.26	2.47	up
PER2	period circadian regulator 2	0.02	0.26	2.03	up
GABRG3	gamma-aminobutyric acid type A receptor gamma3 subunit	0.05	0.31	2.72	up
CATG00000074973.1		0.04	0.30	2.02	up
CABYR	calcium binding tyrosine phosphorylation regulated	0.05	0.31	2.01	up
CATG00000096491.1		0.03	0.28	2.02	up
EFR3B	EFR3 homolog B	0.00	0.24	2.08	up
SSH2	slingshot protein phosphatase 2	0.02	0.26	2.17	up
EP400	E1A binding protein p400	0.02	0.26	2.02	up
SRD5A1	steroid 5 alpha-reductase 1	0.01	0.25	2.42	up
ALX3	ALX homeobox 3	0.00	0.24	2.02	up
GGA3	golgi associated, gamma adaptin ear containing, ARF binding protein 3	0.04	0.29	2.29	up
ZBTB18	zinc finger and BTB domain containing 18	0.02	0.26	2.41	up
STRN3	striatin 3	0.00	0.25	2.11	up
CHD5	chromodomain helicase DNA binding protein 5	0.04	0.29	2.57	up
CHIC1	cysteine rich hydrophobic domain 1	0.00	0.24	2.40	up
SS18L1	SS18L1, nBAF chromatin remodeling complex subunit	0.00	0.24	2.05	up
RIMKLA	ribosomal modification protein rimK like family member A	0.01	0.25	2.03	up
EPHA7	EPH receptor A7	0.02	0.26	2.53	up
IST1	IST1, ESCRT-III associated factor	0.00	0.25	2.20	up
FAT4	FAT atypical cadherin 4	0.02	0.26	2.44	up

TBC1D2B	TBC1 domain family member 2B	0.02	0.26	2.03	up
CLIP1	CAP-Gly domain containing linker protein 1	0.01	0.25	2.05	up
ZC3H4	zinc finger CCCH-type containing 4	0.00	0.24	2.44	up
SCYGR6	small cysteine and glycine repeat containing 6	0.01	0.25	2.03	up
NAB1	NGFI-A binding protein 1	0.01	0.25	2.11	up
ACSL4	acyl-CoA synthetase long chain family member 4	0.03	0.28	2.86	up
PRR14L	proline rich 14 like	0.03	0.28	2.05	up
DENND2A	DENN domain containing 2A	0.00	0.25	2.13	up
PCED1B	PC-esterase domain containing 1B	0.02	0.26	2.50	up
MEF2A	myocyte enhancer factor 2A	0.00	0.25	2.58	up
POLN	DNA polymerase nu	0.00	0.25	2.14	up
SENP5	SUMO specific peptidase 5	0.00	0.24	2.08	up
TMEM245	transmembrane protein 245	0.01	0.25	2.07	up
RASGEF1B	RasGEF domain family member 1B	0.02	0.26	2.16	up
ZNF266	zinc finger protein 266	0.01	0.25	3.27	up
SCML1	Scm polycomb group protein like 1	0.01	0.25	2.16	up
GABRA2	gamma-aminobutyric acid type A receptor alpha2 subunit	0.02	0.26	2.12	up
HOXC13	homeobox C13	0.01	0.25	2.65	up
USPL1	ubiquitin specific peptidase like 1	0.01	0.25	2.46	up
ZNF362	zinc finger protein 362	0.00	0.24	2.12	up
SPTB	spectrin beta, erythrocytic	0.03	0.28	2.16	up
DISP2	dispatched RND transporter family member 2	0.02	0.26	2.29	up
CATG00000060036.1		0.01	0.25	2.15	up
DDX42	DEAD-box helicase 42	0.04	0.29	2.05	up
GAN	gigaxonin	0.00	0.24	2.22	up
ALG10B	ALG10B, alpha-1,2-glucosyltransferase	0.00	0.25	2.06	up
PCDHB8	protocadherin beta 8	0.00	0.24	2.03	up
SNRK	SNF related kinase	0.01	0.25	2.76	up
SEPT12	septin 12	0.03	0.28	2.41	up
CCSER1	coiled-coil serine rich protein 1	0.01	0.25	2.24	up
ARFGEF1	ADP ribosylation factor guanine nucleotide exchange factor 1	0.01	0.25	2.06	up
CSRNP3	cysteine and serine rich nuclear protein 3	0.00	0.24	2.38	up
KBTBD2	kelch repeat and BTB domain containing 2	0.00	0.24	2.13	up
AC003002.3	novel transcript	0.00	0.24	2.01	up
CATG00000113699.1		0.02	0.26	2.06	up
PTPDC1	protein tyrosine phosphatase domain containing 1	0.01	0.25	2.05	up
MAPK8IP3	mitogen-activated protein kinase 8 interacting protein 3	0.02	0.26	2.31	up
ALKBH8	alkB homolog 8, tRNA methyltransferase	0.03	0.28	2.26	up
MBD3L1	methyl-CpG binding domain protein 3 like 1	0.03	0.28	2.24	up
PDXDC1	pyridoxal dependent decarboxylase domain containing 1	0.03	0.28	2.13	up
PRR35	proline rich 35	0.03	0.28	2.80	up
ANK3	ankyrin 3	0.04	0.29	2.01	up
KCNB2	potassium voltage-gated channel subfamily B member 2	0.04	0.29	2.01	up
SLC16A14	solute carrier family 16 member 14	0.01	0.25	2.42	up
RORB	RAR related orphan receptor B	0.00	0.24	2.45	up
BROX	BRO1 domain and CAAX motif containing	0.01	0.25	2.17	up
SEMA4C	semaphorin 4C	0.01	0.25	2.34	up
CNTN1	contactin 1	0.04	0.29	2.06	up
PCDHB11	protocadherin beta 11	0.00	0.24	2.02	up
AASDHPPT	aminoadipate-semialdehyde dehydrogenase-phosphopantetheinyl transferase	0.00	0.24	2.02	up
ZNF852	zinc finger protein 852	0.02	0.27	2.02	up
MAPK8IP2	mitogen-activated protein kinase 8 interacting protein 2	0.02	0.26	2.11	up

HSDL1	hydroxysteroid dehydrogenase like 1	0.00	0.24	2.30	up
SLITRK1	SLIT and NTRK like family member 1	0.01	0.25	3.11	up
PLXNC1	plexin C1	0.01	0.25	2.15	up
ATP6V0A1	ATPase H+ transporting V0 subunit a1	0.02	0.26	2.54	up
SFMBT2	Scm like with four mbt domains 2	0.01	0.25	2.10	up
DPYSL2	dihydropyrimidinase like 2	0.01	0.25	2.03	up
TTPAL	alpha tocopherol transfer protein like	0.00	0.24	2.28	up
PEX5L	peroxisomal biogenesis factor 5 like	0.05	0.31	2.36	up
TBC1D32	TBC1 domain family member 32	0.01	0.25	2.19	up
TERF2	telomeric repeat binding factor 2	0.02	0.26	2.27	up
CHRFAM7A	CHRNA7 (exons 5-10) and FAM7A (exons A-E) fusion	0.00	0.24	2.38	up
L1CAM	L1 cell adhesion molecule	0.03	0.28	2.98	up
GREM2	gremlin 2, DAN family BMP antagonist	0.03	0.28	2.35	up
KCNS2	potassium voltage-gated channel modifier subfamily S member 2	0.02	0.26	2.59	up
ATP7A	ATPase copper transporting alpha	0.01	0.25	2.69	up
HDAC2	histone deacetylase 2	0.01	0.25	2.74	up
DGKD	diacylglycerol kinase delta	0.01	0.25	2.17	up
CPNE5	copine 5	0.03	0.28	2.27	up
ZNF316	zinc finger protein 316	0.02	0.26	2.07	up
AC003006.1	novel protein	0.02	0.26	2.37	up
CATG00000057988.1		0.00	0.19	2.15	up
GPR88	G protein-coupled receptor 88	0.00	0.24	2.11	up
RPS6KA3	ribosomal protein S6 kinase A3	0.01	0.25	2.07	up
SPHKAP	SPHK1 interactor, AKAP domain containing	0.03	0.28	3.82	up
DRD1	dopamine receptor D1	0.02	0.26	2.39	up
FAM234B	family with sequence similarity 234 member B	0.02	0.26	2.24	up
CXorf56	chromosome X open reading frame 56	0.01	0.25	2.27	up
MYCBP2	MYC binding protein 2, E3 ubiquitin protein ligase	0.03	0.28	2.33	up
RASGRF1	Ras protein specific guanine nucleotide releasing factor 1	0.04	0.29	2.00	up
CCDC117	coiled-coil domain containing 117	0.02	0.27	2.17	up
CATG00000024020.1		0.03	0.28	2.09	up
COL25A1	collagen type XXV alpha 1 chain	0.01	0.25	2.32	up
VSNL1	visinin like 1	0.05	0.30	3.83	up
HELQ	helicase, POLQ like	0.02	0.26	2.10	up
DOCK4	dedicator of cytokinesis 4	0.03	0.29	2.03	up
ZXDA	zinc finger X-linked duplicated A	0.02	0.27	2.11	up
GUCY1A2	guanylate cyclase 1 soluble subunit alpha 2	0.02	0.26	2.02	up
GPD1L	glycerol-3-phosphate dehydrogenase 1 like	0.02	0.26	2.03	up
ERCC6	ERCC excision repair 6, chromatin remodeling factor	0.00	0.24	2.71	up
NCOA1	nuclear receptor coactivator 1	0.02	0.27	2.08	up
FAM149A	family with sequence similarity 149 member A	0.01	0.25	2.85	up
EN2	engrailed homeobox 2	0.01	0.25	2.10	up
PRKAA2	protein kinase AMP-activated catalytic subunit alpha 2	0.00	0.24	2.11	up
STK10	serine/threonine kinase 10	0.00	0.24	2.04	up
GSG1L	GSG1 like	0.05	0.31	2.65	up
DOC2B	double C2 domain beta	0.01	0.25	2.66	up
IKZF5	IKAROS family zinc finger 5	0.01	0.25	2.16	up
KIF5A	kinesin family member 5A	0.02	0.26	2.30	up
DRD5	dopamine receptor D5	0.00	0.24	2.64	up
C5orf47	chromosome 5 open reading frame 47	0.04	0.29	2.45	up
CACNG6	calcium voltage-gated channel auxiliary subunit gamma 6	0.00	0.24	2.32	up
B3GALT1	beta-1,3-galactosyltransferase 1	0.00	0.25	2.49	up
CATG00000040194.1		0.01	0.25	2.60	up

WASF1	WAS protein family member 1	0.01	0.25	2.07	up
TRAPPC11	trafficking protein particle complex 11	0.01	0.25	2.11	up
KCNV1	potassium voltage-gated channel modifier subfamily V member 1	0.02	0.26	2.09	up
PRRT4	proline rich transmembrane protein 4	0.05	0.31	2.15	up
PPFIA3	PTPRF interacting protein alpha 3	0.00	0.24	2.32	up
DNM1	dynamin 1	0.04	0.29	2.08	up
FRMD5	FERM domain containing 5	0.00	0.25	2.33	up
MADD	MAP kinase activating death domain	0.01	0.25	3.04	up
APCS	amyloid P component, serum	0.00	0.24	3.90	down
LGALS12	galectin 12	0.03	0.28	2.24	down
UBD	ubiquitin D	0.02	0.25	2.11	down
FAIM	Fas apoptotic inhibitory molecule	0.03	0.28	2.37	down
SOX14	SRY-box 14	0.05	0.31	2.51	down
KRTAP9-7	keratin associated protein 9-7	0.01	0.25	2.30	down
C5orf63	chromosome 5 open reading frame 63	0.00	0.25	3.49	down
CATG00000096311.1		0.01	0.25	2.66	down
GRAP2	GRB2 related adaptor protein 2	0.00	0.25	3.72	down
CBWD1	COBW domain containing 1	0.01	0.25	2.21	down
CATG00000049898.1		0.02	0.26	2.56	down
FCGR1B	Fc fragment of IgG receptor Ib	0.03	0.28	2.07	down
C11orf94	chromosome 11 open reading frame 94	0.04	0.29	2.24	down
PAGE4	PAGE family member 4	0.04	0.29	2.24	down
CLEC19A	C-type lectin domain containing 19A	0.05	0.31	2.08	down
TMEM173	transmembrane protein 173	0.03	0.27	2.21	down
CATG00000111842.1		0.04	0.29	2.72	down
ACTBL2	actin, beta like 2	0.02	0.26	2.69	down
TMCO6	transmembrane and coiled-coil domains 6	0.01	0.25	2.24	down
LRRN4	leucine rich repeat neuronal 4	0.05	0.31	2.03	down
NUPL2	nucleoporin like 2	0.04	0.29	2.09	down
OR2A12	olfactory receptor family 2 subfamily A member 12	0.05	0.31	2.17	down
CDCP2	CUB domain containing protein 2	0.04	0.30	2.08	down
GAD1	glutamate decarboxylase 1	0.02	0.26	2.28	down
PAOX	polyamine oxidase	0.04	0.30	2.35	down
FANCD2	FA complementation group D2	0.03	0.29	2.23	down
SPTBN2	spectrin beta, non-erythrocytic 2	0.01	0.25	2.67	down
KRTAP4-11	keratin associated protein 4-11	0.02	0.26	2.07	down
CTGF	connective tissue growth factor	0.02	0.26	2.11	down
DYRK1A	dual specificity tyrosine phosphorylation regulated kinase 1A	0.04	0.30	2.04	down
CER1	cerberus 1, DAN family BMP antagonist	0.01	0.25	2.43	down
AL035078.4	novel protein	0.03	0.29	2.07	down
NVL	nuclear VCP-like	0.04	0.29	2.09	down
CATG00000001353.1		0.01	0.25	2.21	down
AC096644.1		0.04	0.29	2.91	down
OLR1	oxidized low density lipoprotein receptor 1	0.04	0.30	2.11	down
RXFP1	relaxin family peptide receptor 1	0.01	0.25	2.54	down

Note: false discovery rate (FDR)
