# Supplementary material for: Modeling alcohol-induced neurotoxicity using human induced pluripotent stem cell-derived three-dimensional cerebral organoids
Source: Transl Psychiatry. 2020 Oct 13;10:347. doi: 10.1038/s41398-020-01029-4 (PMC7553959; doi:10.1038/s41398-020-01029-4)
Supplement: Supplementary file 4 — Supplemental Table 2 [file 41398_2020_1029_MOESM4_ESM.pdf]

Supplemental table 2. The reported roles of dysregulated genes in neurodevelopment, nervous system physiology, and neurodegeneration

**Gene Symbol**	**Description of genes**	**Experimental Models**	**Role in neurodevelopment and/or neurodegeneration**	**Reference**
ADAM21	ADAM metallopeptidase domain 21	rodent central nervous system (CNS)	ADAM21 is associated with neurogenesis and axonal growth in post-natal development	[1]
TBX1	T-box	mice	TBX1 encodes transcription factors specifically implicated in cardiac neural-crest-related developmental defects	[2]
PDGFC	platelet derived growth factor	mice	PDGFC is expressed in cerebellum and neurogenic derivatives of neural crest cells; palate formation, craniofacial structures, neural tube, and mesodermal organs	[3] [4]
KCNC1	potassium voltage-gated channel subfamily C member 1	whole-exome sequencing from diagnosed patients	KCNC1 mediates the voltage-dependent potassium ion permeability of excitable membranes, variants prevent neuronal inhibition, which is associated with epilepsy, ataxia, intellectual disability, and developmental delay	[5]
WNT7A	Wnt family member 7A	genetic mouse models, mouse embryos	WNT7A is crucial to the development of the cerebral cortex, synapse formation, and central nervous system vasculature formation and maintenance	[6]
MEF2C	myocyte enhancer factor 2C	mice	MEF2C is highly expressed in developing cortical excitatory neurons, variants linked to autism, intellectual	[7]

			disability, and schizophrenia	
TLE1	transducin like enhancer of split 1	mice	TLE1 inhibits differentiation of neural progenitor cells into neurons, necessary for the maintenance of neuronal survival	[8]
HSD11B2	hydroxysteroid 11-beta dehydrogenase 2	zebrafish	HSD11B2 is involved in stressor-mediated cortisol regulation and protecting glucocorticoid-sensitive tissues during stress	[9]
TUSC1	tumor suppressor candidate 1	Glioblastomas tissues and cell lines	TUSC1 inhibits malignant brain tumor proliferation and induces G1 phases arrest	[10]
KIRREL3	kirre like nephrin family adhesion molecule 3	mice	KIRREL3 is required for the formation of hippocampal mossy fiber synapses, which regulate inhibition of cornu ammonis 3 (CA3) neurons, variants associated with intellectual disabilities	[11]
SHPRH	SNF2 histone linker PHD RING helicase	diagnosed glioma samples and normal brain tissue	SHPRH found abundantly in the human brain, ubiquitinates proliferating cell nuclear antigen, leading to inhibited cell proliferation and tumorigenicity	[12]
AKAIN1	A-kinase anchor inhibitor 1	Neuro2a mouse neuronal cell line	AKAIN1 is expressed preferentially in neural tissues and serves as a disruptor peptide for PKA-AKAP interactions	[13]
PER2	period circadian regulator 2	UK biobank dataset	PER2 is centrally involved with generating and maintaining circadian daily rhythms	[14]
GABRG3	gamma-aminobutyric acid type A receptor gamma3 subunit	genetic mouse models	GABRB3 is important for neurodevelopment, mutations are associated with epilepsy, autism, and visual defects including	[15]

			ocular hypopigmentation	
CABYR	calcium binding tyrosine phosphorylation regulated	Yeast cloning and DNA sequencing	CABYR plays a primary role in the fibrous sheath but is also expressed more distinctly in fetal than the adult brain, providing a potential for a role in brain development	[16]
EP400	E1A binding protein p400	mice	EP400 is essential in the myelination of axons in the central and peripheral nervous systems	[17]
ALX3	ALX homeobox 3	mouse embryos	ALX3 encourages cell differentiation and is involved in cranial neural tube closure, a possible role of patterning the mesoderm during development	[18]
GGA3	golgi associated, gamma adaptin ear containing, ARF binding protein 3	Neuro2A cells	GGA3 regulates trafficking and degradation of enzymes from the Golgi, including some involved in Parkinson's disease and other age-related neurodegenerative disorders	[19]
ZBTB18	zinc finger and BTB domain containing 18	HeLa, HEK293, and P19 embryo-carcinoma cell lines	ZBTB18 encodes DNA-binding transcription factors essential for myogenesis and brain development, variants can lead to brain developmental disease	[20]
CHD5	chromodomain helicase DNA binding protein 5	rodent cerebral cortex	CHD5 plays a role in the development of the nervous system by activating the expression of genes promoting neuron terminal differentiation	[21]
SS18L1	SS18L1, nBAF chromatin remodeling	genetic mouse models	SS18L1 encodes calcium-responsive transactivator (CREST),	[22]

	complex subunit		which when deleted leads to neuroinflammation and ALS-like motor defects	
RIMKLA	ribosomal modification protein rimK like family member A	murine brain tissue and sciatic nerves	RIMKLA synthesizes N-acetyl-L-aspartyl-L-glutamate (NAAG), found in sciatic nerves, spinal, cord, and brainstem and predicted to act as a neurotransmitter	[23]
EPHA7	EPH receptor A7	adult rat and mouse brain	EPHA7 regulates brain development modulating cell-cell adhesion and repulsion and plays key roles in adult CNS synaptic maintenance, plasticity, and function	[24]
FAT4	FAT atypical cadherin 4	complete coding sequence of FAT4_human	FAT4 acts as a positive regulator of planar cell polarity and is expressed in fetal brain, infant brain, brain tumor, and colorectal cancer	[25]
NAB1	NGFI-A binding protein 1	C. elegans ventral nerve cord	NAB1 can interact with SYD1 in absence of Rac proteins to inhibit axon outgrowth and prohibit neurite development	[26]
DENND2A	DENN domain containing 2A	Ischemic stroke and Parkinson's Disease patient tissue and whole blood samples	DENND2A acts as a specific guanine nucleotide exchange factor (GEF) for Rab9, possible role in mitophagy-determined etiology of Parkinson’s disease and ischemic stroke	[27]
PCED1B	PC-esterase domain containing 1B	Human astrocyte and glioma cells	PCED1B knockdown restricted cell proliferation and induced cell apoptosis in glioma cells	[28]
MEF2A	myocyte enhancer factor 2A	human and rat temporal neocortex tissue	MEF2A regulates synaptogenesis, dendritic morphogenesis, and neuronal survival, and is	[29]

			found to be downregulated in temporal lobe epilepsy	
SENP5	SUMO specific peptidase 5	murine brain tissue	SENP5 in cortical neurons is localized to presynaptic terminals, postsynaptic spines, and mitochondria in axon terminals, suggesting a role for SUMOylation in synaptic regulation	[30]
RASGEF1B	RasGEF domain family member 1B	mouse embryos	RASGEF1B is expressed primarily in cortical interneurons in the cortical subventricular zone and may play a role in tangential migration of interneurons	[31]
GABRA2	gamma-aminobutyric acid type A receptor alpha2 subunit	genetic and clinical data of diagnosed patients	GABRA2 variants are associated with early-onset epileptic encephalopathy and intellectual disability with seizures	[32]
HOXC13	homeobox C13	mice brain microvascular endothelial cells; sheep embryonic mRNA	HOXC13 is upregulated by vascular endothelial growth factor (VEGF) in vitro models of cerebral ischemia, aggravating the blood brain barrier; indicated in the development of hair, nail, and filiform papilla	[33]
USPL1	ubiquitin specific peptidase like 1	SMN2 mice embryos and neonates	USPL1 changes in alternative splicing found in phenotypic-stage severe spinal muscular atrophy embryo and neonate models and these changes became more pronounced with the severity of the disease	[34]
SPTB	spectrin beta, erythrocytic	patient with diagnosed autism and spherocytosis	SPTB encodes a cytoskeleton protein previously associated with spherocytosis, deleted in case studies with	[35]

			spherocytosis, severe learning disabilities, and mild mental retardation	
DISP2	dispatched RND transporter family member 2	zebrafish	DISP2 is expressed in the telencephalon and ventral hindbrain in zebrafish found to be essential for normal embryonic development	[36]
GAN	gigaxonin	diagnosed giant axonal neuropathy patients	GAN synonymous mutations and intragenic deletions are involved in giant axonal neuropathy, an age-related progressive neurodegenerative disease involving PNS and CNS	[37]
PCDHB8	protocadherin beta 8	Ms5Yah mouse model	PCDHB8 is disrupted in genetic mouse models of partial monosomy 21, characterized by intellectual disabilities involving spatial learning and memory impairments, craniofacial dysmorphology, short stature, and muscular and heart defects	[38]
SNRK	SNF related kinase	rat cerebellar granule neurons	SNRK is implicated in the regulation of low K+ induced apoptosis of cerebellar granule neurons	[39]
CCSER1	coiled-coil serine rich protein 1	Parkinson's patient	CCSER1 is disrupted at the genomic breakpoint in early-onset, sever parkinsonism-dementia syndrome	[40]
ARFGEF1	ADP ribosylation factor guanine nucleotide exchange factor 1	genetic mouse models	ARFGEF1 haploinsufficiency is associated with developmental delay and decreased threshold to spontaneous seizure activity suggested to impair neuronal inhibition	[41]

CSRNP3	cysteine and serine rich nuclear protein 3	unigene libraries of mouse tissues	CSRNP3 homologue is only expressed in the mouse brain and spinal cord during embryonic development but in all regions of the adult brain	[42]
MAPK8IP3	mitogen-activated protein kinase 8 interacting protein 3	MAPK8IP3 variant case studies, zebrafish embryos	MAPK8IP3 variants are associated with spastic diplegia, intellectual disability, cerebral atrophy, and corpus callosum hypoplasia; overexpression in zebrafish embryos had an adverse effect on developing axons	[43]
ALKBH8	alkB homolog 8, tRNA methyltransferase	tRNA of ALKBH8 mutated individuals	ALKBH8 truncations affect wobble modifications, which the brain is sensitive to and is associated with intellectual disability	[44]
PDXDC1	pyridoxal dependent decarboxylase domain containing 1	genetic mouse models	PDXDC1 suppression increases acoustic prepulse inhibition, a schizophrenia endophenotype, and is highly expressed in the hippocampus	[45]
ANK3	ankyrin 3	genetic mouse models	ANK3 suppression is linked to schizophrenia, bipolar disorder, intellectual disability, and autism spectrum disorders, and heterozygous knockouts in mice show smaller cingulate cortex, granular retrosplenial cortex, primary motor cortex, and fimbria of the hippocampus	[46]
KCNB2	potassium voltage-gated channel subfamily B	mouse dorsal root ganglion neurons	KCNB2 plays a role in the postnatal maturation of dorsal root ganglion neurons, expression gradually	[47]

	member 2		decreases between 1 and 4 weeks of age	
SLC16A14	solute carrier family 16 member 14	mouse brain tissue	SLC16A14 encodes a protein exclusively localized in the soma of neurons and highly expressed in excitatory and inhibitory neurons and epithelial cells in the mouse brain suggested to be a neuronal aromatic-amino-acid transporter	[48]
RORB	RAR related orphan receptor B	whole exome sequencing from diagnosed patients	RORB variants are associated with neurodevelopmental disorders, especially in generalized epilepsies with predominant absence seizures, and is hypothesized to control cytoarchitectural patterning of neocortical neurons during development	[49]
SEMA4C	semaphorin 4C	genetic mouse models	SEMA4C signaling knockout in mouse forebrain indicates a large role of this gene in the recent and remote recall of fear memory, specifically by enhancing dendritic ramifications and modulating synaptic density in the adult hippocampus	[50]
CNTN1	contactin 1	healthy adult corpus collosum tissue	CNTN1 gene variations are linked to impacted axon density in the corpus collosum, and is suggested to be an axon guidance gene	[51]
PCDHB11	protocadherin beta 11	K562 cells	PCDBH11 is a human-specific brain-expressed protein with homophilic cell adhesion	[52]

			functions	
AASDHPPT	aminoadipate-semialdehyde dehydrogenase-phosphopantetheinyl transferase	human glioma metabolomics	AASDHPPT is a sulfotransferase found up-regulated in malignant brain tumor	[53]
ZNF852	zinc finger protein 852	whole genome analysis of epileptic patients	ZNF852 downregulation was associated with seizure-free outcome of epileptic patients following anterior temporal lobectomy with amygdalohippocampectomy	[54]
MAPK8IP2	mitogen-activated protein kinase 8 interacting protein 2	RNA sequencing of human brain tissue	MAPK8IP2 expression is high throughout fetal life and infancy, and is suggested to enrich genes related to axonal growth and synaptic function during neurodevelopment; deleted in Phelan-McDermid syndrome, characterized by intellectual disability, severely delayed language development, and specific facial features	[55]
SLITRK1	SLIT and NTRK like family member 1	genomic screenings	SLITRK1 variants are associated with obsessive-compulsive disorder and Tourette disorder phenotypes	[56, 57]
PLXNC1	plexin C1	genetic mouse models	PLXNC1 directs axonal guidance of midbrain dopaminergic neurons and the segregation of mesolimbic and nigrostriatal dopaminergic pathways	[58]
ATP6V0A1	ATPase H^+ transporting V0 subunit a1	human microglial cells	ATP6V0A1 elevation attenuates rotenone-induced microglia inflammation, which is implicated in the	[59]

			chronic inflammation of Parkinson’s disease	
DPYSL2	dihydropyrimidinase like 2	genetic mouse models	DPYSL2 is identified as an alcohol-sensitive Alzheimer's Disease-related protein that regulates cytoskeleton development	[60]
CHRFAM7A	CHRNA7 (exons 5-10) and FAM7A (exons A-E) fusion	clinical data of diagnosed patients	CHRFAM7A variation is associated with pro-inflammatory responses, which is significantly linked to elevated pain experience following spinal cord injury	[61]
L1CAM	L1 cell adhesion molecule	genetic rat models	L1CAM gene mutations are associated with X-linked human congenital hydrocephalus and can accelerate early hydrocephalus phenotypes of Ccdc39 knockout rats showing impaired cortical development and glymphatic Cerebrospinal fluid (CSF) flow	[62]
GREM2	gremlin 2, DAN family BMP antagonist	SH-SY5Y neuronal cells	GREM2 effects neuroprotection via downregulation in neuronal cells exposed to cadmium, which is linked to neurotoxicity and neurodegenerative diseases, including Alzheimer's and Parkinson’s	[63]
KCNS2	potassium voltage-gated channel modifier subfamily S member 2	genetic and clinical data of a diagnosed patient	KCNS2, via a microdeletion, is a possible candidate for explaining a complex neurological phenotype involving developmental delay, microcephaly, seizures, and typical facial dysmorphism	[64]

ATP7A	ATPase copper transporting alpha	genetic and clinical data of diagnosed patients	ATP7A mutations have been associated with severe infantile neurodegenerative Menkes diseases and Occipital horn syndrome, a novel missense mutation was identified in the familial presentation of distal motor neuropathy and autonomic dysfunction	[65]
HDAC2	histone deacetylase 2	genetic mouse models	HDAC2 upregulation in dorsal hippocampal neurons resulted in neuroinflammation-induced memory impairment and reduced histone acetylation, and suppression was able to reverse microglial activation and memory deficits	[66]
DGKD	diacylglycerol kinase delta	diagnosed epileptic patient, array Comparative Genomic Hybridization (CGH)	DGKD encodes a cytoplasmic enzyme involved in the production of phosphatic acid, deletion and mutations are associated with epilepsy and other neurological diseases, though the mutation alone is not sufficient to cause epilepsy	[67]
CPNE5	copine 5	genetic mouse models	CPNE5 deficiency in mice does not affect rodent locomotor activity or memory, but significantly decreased anxiety level during stress-inducing tasks, indicating a role of this gene in regulating anxiety level	[68]
GPR88	G protein-coupled receptor 88	genetic rat models	GPR88 knockdown reduced acute amphetamine-induced and increased L-DOPA-induced turning	[69]

			behavior in a rat model of hemiparkinsonism and normalized the upregulated expression of striatal Gad67, and is identified as a possible new target for the management of Parkinson's disease	
RPS6KA3	ribosomal protein S6 kinase A3	genetic and clinical data of diagnosed patients	RPS6KA3 microduplication is identified in several cases of X-linked intellectual disabilities, with carriers displaying major depression and mild intellectual disability	[70]
SPHKAP	SPHK1 interactor, AKAP domain containing	genomic wide association studies of Japanese population	SPHKAP is identified as a susceptibility locus for schizophrenia in the Japanese and Psychiatric Genomic Consortium populations	[71]
DRD1	dopamine receptor D1	prenatally stressed bull calves	DRD1 is associated with behavior and stress response and was found to be differentially methylated in the white blood cells of bull calves who underwent prenatal stress	[72]
FAM234B	family with sequence similarity 234 member B	exome sequencing of diagnosed patients	FAM234B homozygous truncations were identified in 31.6% of families associated with intellectual disability	[73]
CXorf56	chromosome X open reading frame 56	genetic and clinical data of diagnosed patients	CXorf56 reduced expression was identified in one family associated with X-linked intellectual disability of behavioral problems, and is present and located in the nucleus, cell, soma, dendrites, and dendritic spines of the murine brain and primary hippocampal	[74]

			neuron cultures	
MYCBP2	MYC binding protein 2, E3 ubiquitin protein ligase	GWAS, SH-SY5Y cells, and primary neuronal cultures	MYCBP2 induces the ubiquitination of TSC2 and downstream decreases transcription of SYT11, which can induce lysosomal dysfunction and impair degradation of autophagosomes, which is likely to contribute to some forms of Parkinson's disease associated neurodegeneration	[75]
RASGRF1	Ras protein specific guanine nucleotide releasing factor 1	primary cultured cortical neurons	RASGRF1 is an intrinsic key mediator for BDNF-induced R-Rase activation and R-Ras mediated axonal morphological regulation, which is a key enabler of higher brain function in the central nervous system	[76]
COL25A1	collagen type XXV alpha 1 chain	mouse brain	COL25A1 was found to be expressed in mouse retina and specifically enriched in retin-recipient nuclei within the brain, suggesting a role in brain development and function	[77]
VSNL1	visinin like 1	RNAseq on genetic mouse model	VSNL1 increase was found in genetic mouse models of Helios-specific spatial memory deficits, and normalization of VSNL1 was able to reverse their CA30CA1 neuronal spine density loss phenotype	[78]
DOCK4	dedicator of cytokinesis 4	genetic mouse models	DOCK4 knockout mice displayed a series of autism spectrum disorder-like behaviors, and restoration corrected impaired social	[79]

			deficits and social novelty deficits, suggesting a mechanism in regulating hippocampal excitatory synaptic transmission and social behavior	
ERCC6	ERCC excision repair 6, chromatin remodeling factor	genetic and clinical data of diagnosed patients	ERCC6 variants are present in two thirds of individuals with Cackayne syndrome, a disease impacting a spectrum of phenotypes including impaired postnatal growth, progressive impairment of vision, central and peripheral nervous system function that leads to severe disability	[80]
EN2	engrailed homeobox 2	genetic mouse models	EN2 knockout in the excitatory cerebellar nuclei neurons (eCN) leads to reduced postnatal growth of the cerebellar cortex through the loss of the eCN and interactions with Purkinje cells, which regulate the production of granule cells and interneurons	[81]
PRKAA2	protein kinase AMP-activated catalytic subunit alpha 2	genetic mouse models	PRKAA2 disruption significantly decreased apoptosis of neural progenitor cells in mice after irradiation, suggesting a role of this gene in determining cell fate response following DNA damage in NPCs	[82]
GSG1L	GSG1 like	genetic mouse models	GSG1L is found to regulate the strength of AMPA receptor-mediated synaptic transmission in hippocampal dentate granule neurons, suggesting a neuronal-type	[83]

			specific role of this gene	
KIF5A	kinesin family member 5A	genetic and clinical data of diagnosed patients	KIF5A mediates the transport of VAPB, mutations of this function and in the KIF5A gene have been associated with late-onset spinal muscular atrophy	[84]
DRD5	dopamine receptor D5	prenatally stressed bull calves;<br>SK-N-SH cell line	DRD5, much like DRD1, is associated with behavior and stress response, and was found to be differentially methylated in the white blood cells of bull calves who underwent prenatal stress; DRD5 was also found to be upregulated by overexpression of a lncRNA identified in peripheral blood of schizophrenic patients, and downregulated by its knockdown	[72] [85]
CACNG6	calcium voltage-gated channel auxiliary subunit gamma 6	WGAS of the Chinese Han population	CACNG6 contributes to the gamma subunit of voltage-gated L-type calcium channels, with polymorphisms in this gene found to potentially associate with increased risk of schizophrenia, and a strong interaction suggesting a possible biological interaction with CACNG5	[86]
B3GALT1	beta-1,3-galactosyltransferase 1	GWAS on Parkinson's patients	B3GALT1 is contained within a chromosomal region often affected by Parkinson's disease, and variants in this gene have been identified in PD patients but not yet associated with particular risk or phenotype	[87]
WASF1	WAS protein	WGAS of ID	WASF1 truncations have	[88]

	family member 1	patients, function studies of patient fibroblast cells	been identified in unrelated individuals with intellectual disability, autistic features, and seizures, and have been shown to affect the actin remodeling in fibroblasts from these patients	
KCNV1	potassium voltage-gated channel modifier subfamily V member 1	WES and blood cell transcriptome of male autism spectrum disorder (ASD) patients	KCNV1 mutations have been identified in male autism spectrum disorder patients and are associated with an ADHD phenotype, and defects in voltage-gated potassium channels have previously been noted in other neuropsychiatric disorders such as bipolar disorder, ASD, and schizophrenia	[89]
DNM1	dynamin 1	WES in diagnosed patients	DNM1 encodes a protein necessary in the process of neurotransmitter signaling and CNS development, variants have been associated with global developmental delay and severe intellectual disability, and most with epileptic encephalopathy	[90]
APCS	amyloid P component, serum	F57I fly variants	APCS encodes a protein that can prevent cell death in the CNS in diseased flies, and prevent the accumulation of insoluble forms of lysosomes in both WT and diseased flies, suggesting an ability of this protein to prevent cell death by preventing the accumulation of toxic F57I structures	[91]
FAIM	Fas apoptotic	neuronal PC12	FAIM encodes an	[92]

	inhibitory molecule	cells, MM cell lines, genetic data from diagnosed patients	anti-apoptotic protein upregulated in B cell receptor-activated V cells and provides resistance to Fas-mediated cell death, but has also been found to have a function in cell growth, metabolism, Alzheimer's disease, and tumorigenesis	
SOX14	SRY-box 14	mouse cerebellar culture, genetic mouse models	SOX14 has been found to be an exclusive marker of inhibitory projection neurons in the lateral and interposed cerebellar nuclei, targeted ablation of Sox14 positive cells leads to significantly impaired motor learning	[93]
C5orf63	chromosome 5 open reading frame 63	whole transcript expression profiling in meningiomas	C5orf63 was significantly upregulated in meningiomas, or primary brain tumors, classified as DCC (deleted in colorectal cancer) gene low expression compared to high expression	[94]
FCGR1B	Fc fragment of IgG receptor Ib	GIBHi001-A hIPSC line	FCGR1B mutations were found in human induced pluripotent stem cells derived from a patient with autism spectrum disorder and it has a suggested role in the humoral immune response	[95]
TMEM173	transmembrane protein 173	genetic mouse models	TMEM173 encodes a protein that plays a crucial role in modulating information, and knockout is shown to alleviate inflammatory response and facilitate recovery following spinal cord injury in mice	[96]
LRRN4	leucine rich repeat neuronal	mouse embryos	LRRN4 is expressed in dorsal root ganglion (DRG)	[97]

	4		cells of adult mice, expression patterns show increasing rates from embryo to postnatal, then decrease dramatically to adult levels, suggesting a contribution to synaptic formation or development of synaptic function in DRG neurons	
NUPL2	nucleoporin like 2	mouse and human cerebral cortex cells	NUPL2 expression shows regional variability in the brain, with the highest expression being in temporal cortex in normal brain tissue, and after introducing an SNP seen in Parkinson's disease, NUPL2 showed increased expression in cerebellum, temporal cortex, and frontal cortex	[98]
CDCP2	CUB domain containing protein 2	whole genome analysis of epileptic patients	CDCP2 downregulation, much like ZNF852, was associated with seizure-free outcome of epileptic patients following anterior temporal lobectomy with amygdalohippocampectomy	[54]
GAD1	glutamate decarboxylase 1	GAD1 interneurons	GAD1 expressing interneurons block the antidepressant actions of ketamine when GluN2B is knocked down, this prevents an increase in extracellular glutamate and synaptic formation in the prefrontal cortex	[99]
SPTBN2	spectrin beta, non-erythrocytic 2	genetic data of diagnosed patients	SPTBN2 mutations are associated with infantile onset of spinocerebellar ataxia type 5, which presents with ataxic cerebral palsy	[100]

DYRK1A	dual specificity tyrosine phosphorylation regulated kinase 1A	transgenic mouse models	DYRK1A overexpression is associated with cognitive phenotypes of down syndrome and is shown to alter protein and phosphoprotein levels of key postsynaptic and plasticity-related pathways	[101]
NVL	nuclear VCP-like	genetic data of diagnosed patients	NVL is suggested to contain overlapping genetic risk factors for major depressive disorder and schizophrenia in the Han Chinese population	[102]
OLR1	oxidized low density lipoprotein receptor 1	neuroinflammatory mouse model	OLR1 overexpression is found in activated microglia, while silencing of the gene inhibited MAPK phosphorylation, some nuclear transport, and pro-inflammatory factor production in microglia, suggesting a role of OLR1 in aggravating neuroinflammation-induced neuronal apoptosis	[103]

Note: *CNS: central nervous system; PNS: peripheral nervous system; CSF: cerebrospinal fluid; GWAS: genome-wide association studies; NPC: neural progenitor cell; lncRNA: long non-coding RNA; PD: Parkinson's disease; ID: intellectual disability; WES: whole exome sequencing; ASD: Autism spectrum disorders; ADHD: attention deficit hyperactivity disorder; WT: wild type; MM: multiple myeloma; SNP: single nucleotide polymorphism; PKA: protein kinase A; AKAP: a-kinase anchoring protein; ALS: amyotrophic lateral sclerosis; BDNF: brain-derived neurotrophic factor; AMPA: α-amino-3-hydroxy-5-methyl-4-isoxazolepropionic acid; GLUN2B: glutamate ionotropic receptor NMDA type subunit 2B; MAPK: mitogen-activated protein kinase*

References

1. Yang P, Baker KA, Hagg T. A disintegrin and metalloprotease 21 (ADAM21) is associated with neurogenesis and axonal growth in developing and adult rodent CNS. *J Comp Neurol* 2005; **490**(2)**:** 163-179.
2. Calmont A, Ivins S, Van Bueren KL, Papangeli I, Kyriakopoulou V, Andrews WD *et al.* Tbx1 controls cardiac neural crest cell migration during arch artery development by regulating Gbx2 expression in the pharyngeal ectoderm. *Development* 2009; **136**(18)**:** 3173-3183.
3. Ding H, Wu X, Kim I, Tam PP, Koh GY, Nagy A. The mouse Pdgfc gene: dynamic expression in embryonic tissues during organogenesis. *Mech Dev* 2000; **96**(2)**:** 209-213.
4. Ding H, Wu X, Boström H, Kim I, Wong N, Tsoi B *et al.* A specific requirement for PDGF-C in palate formation and PDGFR-alpha signaling. *Nat Genet* 2004; **36**(10)**:** 1111-1116.

5. Park J, Koko M, Hedrich UBS, Hermann A, Cremer K, Haberlandt E *et al.* KCNC1-related disorders: new de novo variants expand the phenotypic spectrum. *Ann Clin Transl Neurol* 2019; **6**(7)**:** 1319-1326.
6. Lan L, Wang W, Huang Y, Bu X, Zhao C. Roles of Wnt7a in embryo development, tissue homeostasis, and human diseases. *J Cell Biochem* 2019; **120**(11)**:** 18588-18598.
7. Harrington AJ, Raissi A, Rajkovich K, Berto S, Kumar J, Molinaro G *et al.* MEF2C regulates cortical inhibitory and excitatory synapses and behaviors relevant to neurodevelopmental disorders. *Elife* 2016; **5**.
8. Dastidar SG, Narayanan S, Stifani S, D'Mello SR. Transducin-like enhancer of Split-1 (TLE1) combines with Forkhead box protein G1 (FoxG1) to promote neuronal survival. *J Biol Chem* 2012; **287**(18)**:** 14749-14759.
9. Alderman SL, Vijayan MM. 11β-Hydroxysteroid dehydrogenase type 2 in zebrafish brain: a functional role in hypothalamus-pituitary-interrenal axis regulation. *J Endocrinol* 2012; **215**(3)**:** 393-402.
10. Zhang R, Yu W, Liang G, Jia Z, Chen Z, Zhao L *et al.* Tumor Suppressor Candidate 1 Suppresses Cell Growth and Predicts Better Survival in Glioblastoma. *Cell Mol Neurobiol* 2017; **37**(1)**:** 37-42.
11. Martin EA, Muralidhar S, Wang Z, Cervantes DC, Basu R, Taylor MR *et al.* The intellectual disability gene Kirrel3 regulates target-specific mossy fiber synapse development in the hippocampus. *Elife* 2015; **4:** e09395.
12. Zhang M, Huang N, Yang X, Luo J, Yan S, Xiao F *et al.* A novel protein encoded by the circular form of the SHPRH gene suppresses glioma tumorigenesis. *Oncogene* 2018; **37**(13)**:** 1805-1814.
13. Fukuda M, Aizawa Y. Hypothetical gene C18orf42 encodes a novel protein kinase A-binding protein. *Genes Cells* 2015; **20**(4)**:** 267-280.
14. Chang AM, Duffy JF, Buxton OM, Lane JM, Aeschbach D, Anderson C *et al.* Chronotype Genetic Variant in PER2 is Associated with Intrinsic Circadian Period in Humans. *Sci Rep* 2019; **9**(1)**:** 5350.
15. Delahanty RJ, Zhang Y, Bichell TJ, Shen W, Verdier K, Macdonald RL *et al.* Beyond Epilepsy and Autism: Disruption of GABRB3 Causes Ocular Hypopigmentation. *Cell Rep* 2016; **17**(12)**:** 3115-3124.
16. Hsu HC, Lee YL, Cheng TS, Howng SL, Chang LK, Lu PJ *et al.* Characterization of two non-testis-specific CABYR variants that bind to GSK3beta with a proline-rich extensin-like domain. *Biochem Biophys Res Commun* 2005; **329**(3)**:** 1108-1117.
17. Fröb F, Sock E, Tamm ER, Saur AL, Hillgärtner S, Williams TJ *et al.* Ep400 deficiency in Schwann cells causes persistent expression of early developmental regulators and peripheral neuropathy. *Nat Commun* 2019; **10**(1)**:** 2361.
18. Lakhwani S, García-Sanz P, Vallejo M. Alx3-deficient mice exhibit folic acid-resistant craniofacial midline and neural tube closure defects. *Dev Biol* 2010; **344**(2)**:** 869-880.

19. von Einem B, Eschbach J, Kiechle M, Wahler A, Thal DR, McLean PJ *et al.* The Golgi-localized, gamma ear-containing, ARF-binding (GGA) protein family alters alpha synuclein (α-syn) oligomerization and secretion. *Aging (Albany NY)* 2017; **9**(7)**:** 1677-1697.
20. Hemming IA, Clément O, Gladwyn-Ng IE, Cullen HD, Ng HL, See HB *et al.* Disease-associated missense variants in ZBTB18 disrupt DNA binding and impair the development of neurons within the embryonic cerebral cortex. *Hum Mutat* 2019; **40**(10)**:** 1841-1855.
21. Goodman JV, Bonni A. Regulation of neuronal connectivity in the mammalian brain by chromatin remodeling. *Curr Opin Neurobiol* 2019; **59:** 59-68.
22. Cheng C, Yang K, Wu X, Zhang Y, Shan S, Gitler A *et al.* Loss of CREST leads to neuroinflammatory responses and ALS-like motor defects in mice. *Transl Neurodegener* 2019; **8:** 13.
23. Lodder-Gadaczek J, Becker I, Gieselmann V, Wang-Eckhardt L, Eckhardt M. N-acetylaspartylglutamate synthetase II synthesizes N-acetylaspartylglutamylglutamate. *J Biol Chem* 2011; **286**(19)**:** 16693-16706.
24. Amegandjin CA, Jammow W, Laforest S, Riad M, Baharnoori M, Badeaux F *et al.* Regional expression and ultrastructural localization of EphA7 in the hippocampus and cerebellum of adult rat. *J Comp Neurol* 2016; **524**(12)**:** 2462-2478.
25. Katoh Y, Katoh M. Comparative integromics on FAT1, FAT2, FAT3 and FAT4. *Int J Mol Med* 2006; **18**(3)**:** 523-528.
26. Nørgaard S, Deng S, Cao W, Pocock R. Distinct CED-10/Rac1 domains confer context-specific functions in development. *PLoS Genet* 2018; **14**(9)**:** e1007670.
27. Lang W, Wang J, Ma X, Zhang N, Li H, Cui P *et al.* Identification of Shared Genes Between Ischemic Stroke and Parkinson's Disease Using Genome-Wide Association Studies. *Front Neurol* 2019; **10:** 297.
28. Yang J, Yu D, Liu X, Changyong E, Yu S. LncRNA PCED1B-AS1 activates the proliferation and restricts the apoptosis of glioma through cooperating with miR-194-5p/PCED1B axis. *J Cell Biochem* 2019.
29. Huang Y, Wu X, Guo J, Yuan J. Myocyte-specific enhancer binding factor 2A expression is downregulated during temporal lobe epilepsy. *Int J Neurosci* 2016; **126**(9)**:** 786-796.
30. Akiyama H, Nakadate K, Sakakibara SI. Synaptic localization of the SUMOylation-regulating protease SENP5 in the adult mouse brain. *J Comp Neurol* 2018; **526**(6)**:** 990-1005.
31. Tao G, Li Z, Wen Y, Song X, Wei S, Du H *et al.* Transcription Factors. *Front Mol Neurosci* 2019; **12:** 75.
32. Maljevic S, Keren B, Aung YH, Forster IC, Mignot C, Buratti J *et al.* Novel GABRA2 variants in epileptic encephalopathy and intellectual disability with seizures. *Brain* 2019; **142**(5)**:** e15.

33. Sulayman A, Tian K, Huang X, Tian Y, Xu X, Fu X *et al.* Genome-wide identification and characterization of long non-coding RNAs expressed during sheep fetal and postnatal hair follicle development. *Sci Rep* 2019; **9**(1)**:** 8501.
34. Liu H, Shafey D, Moores JN, Kothary R. Neurodevelopmental consequences of Smn depletion in a mouse model of spinal muscular atrophy. *J Neurosci Res* 2010; **88**(1)**:** 111-122.
35. Griswold AJ, Ma D, Sacharow SJ, Robinson JL, Jaworski JM, Wright HH *et al.* A de novo 1.5 Mb microdeletion on chromosome 14q23.2-23.3 in a patient with autism and spherocytosis. *Autism Res* 2011; **4**(3)**:** 221-227.
36. Li N, Flynt AS, Kim HR, Solnica-Krezel L, Patton JG. Dispatched Homolog 2 is targeted by miR-214 through a combination of three weak microRNA recognition sites. *Nucleic Acids Res* 2008; **36**(13)**:** 4277-4285.
37. Echaniz-Laguna A, Cuisset JM, Guyant-Marechal L, Aubourg P, Kremer L, Baaloul N *et al.* Giant axonal neuropathy: a multicenter retrospective study with genotypic spectrum expansion. *Neurogenetics* 2019.
38. Arbogast T, Raveau M, Chevalier C, Nalesso V, Dembele D, Jacobs H *et al.* Deletion of the App-Runx1 region in mice models human partial monosomy 21. *Dis Model Mech* 2015; **8**(6)**:** 623-634.
39. Yoshida K, Yamada M, Nishio C, Konishi A, Hatanaka H. SNRK, a member of the SNF1 family, is related to low K(+)-induced apoptosis of cultured rat cerebellar granule neurons. *Brain Res* 2000; **873**(2)**:** 274-282.
40. Zafar F, Valappil RA, Kim S, Johansen KK, Chang ALS, Tetrud JW *et al.* Genetic fine-mapping of the Iowan. *NPJ Parkinsons Dis* 2018; **4:** 18.
41. Teoh J, Subramanian N, Pero ME, Bartolini F, Amador A, Kanber A *et al.* Arfgef1 haploinsufficiency in mice alters neuronal endosome composition and decreases membrane surface postsynaptic GABA. *Neurobiol Dis* 2019; **134:** 104632.
42. Yang HL, Cho EY, Han KH, Kim H, Kim SJ. Characterization of a novel mouse brain gene (mbu-1) identified by digital differential display. *Gene* 2007; **395**(1-2)**:** 144-150.
43. Iwasawa S, Yanagi K, Kikuchi A, Kobayashi Y, Haginoya K, Matsumoto H *et al.* Recurrent de novo MAPK8IP3 variants cause neurological phenotypes. *Ann Neurol* 2019; **85**(6)**:** 927-933.
44. Monies D, Vågbø CB, Al-Owain M, Alhomaidi S, Alkuraya FS. Recessive Truncating Mutations in ALKBH8 Cause Intellectual Disability and Severe Impairment of Wobble Uridine Modification. *Am J Hum Genet* 2019; **104**(6)**:** 1202-1209.
45. Feldcamp LA, Boutros PC, Raymond R, Fletcher PJ, Nobrega JN, Wong AHC. Pdxdc1 modulates prepulse inhibition of acoustic startle in the mouse. *Transl Psychiatry* 2017; **7**(5)**:** e1125.
46. van der Werf IM, Van Dam D, Missault S, Yalcin B, De Deyn PP, Vandeweyer G *et al.* Behavioural characterization of AnkyrinG deficient mice, a model for ANK3 related disorders. *Behav Brain Res* 2017; **328:** 218-226.

47. Regnier G, Bocksteins E, Van de Vijver G, Snyders DJ, van Bogaert PP. The contribution of Kv2.2-mediated currents decreases during the postnatal development of mouse dorsal root ganglion neurons. *Physiol Rep* 2016; **4**(6).
48. Roshanbin S, Lindberg FA, Lekholm E, Eriksson MM, Perland E, Åhlund J *et al.* Histological characterization of orphan transporter MCT14 (SLC16A14) shows abundant expression in mouse CNS and kidney. *BMC Neurosci* 2016; **17**(1)**:** 43.
49. Rudolf G, Lesca G, Mehrjouy MM, Labalme A, Salmi M, Bache I *et al.* Loss of function of the retinoid-related nuclear receptor (RORB) gene and epilepsy. *Eur J Hum Genet* 2016; **24**(12)**:** 1761-1770.
50. Simonetti M, Paldy E, Njoo C, Bali KK, Worzfeld T, Pitzer C *et al.* The impact of Semaphorin 4C/Plexin-B2 signaling on fear memory via remodeling of neuronal and synaptic morphology. *Mol Psychiatry* 2019.
51. Anderson C, Gerding WM, Fraenz C, Schlüter C, Friedrich P, Raane M *et al.* PLP1 and CNTN1 gene variation modulates the microstructure of human white matter in the corpus callosum. *Brain Struct Funct* 2018; **223**(8)**:** 3875-3887.
52. de Freitas GB, Gonçalves RA, Gralle M. Functional test of PCDHB11, the most human-specific neuronal surface protein. *BMC Evol Biol* 2016; **16:** 75.
53. Li W, Jia H, Li Q, Cui J, Li R, Zou Z *et al.* Glycerophosphatidylcholine PC(36:1) absence and 3'-phosphoadenylate (pAp) accumulation are hallmarks of the human glioma metabolome. *Sci Rep* 2018; **8**(1)**:** 14783.
54. Gallek MJ, Skoch J, Ansay T, Behbahani M, Mount D, Manziello A *et al.* Cortical gene expression: prognostic value for seizure outcome following temporal lobectomy and amygdalohippocampectomy. *Neurogenetics* 2016; **17**(4)**:** 211-218.
55. Ziats CA, Grosvenor LP, Sarasua SM, Thurm AE, Swedo SE, Mahfouz A *et al.* Functional genomics analysis of Phelan-McDermid syndrome 22q13 region during human neurodevelopment. *PLoS One* 2019; **14**(3)**:** e0213921.
56. Melo-Felippe FB, Fontenelle LF, Kohlrausch FB. Gene variations in PBX1, LMX1A and SLITRK1 are associated with obsessive-compulsive disorder and its clinical features. *J Clin Neurosci* 2019; **61:** 180-185.
57. Fernandez TV, State MW, Pittenger C. Tourette disorder and other tic disorders. *Handb Clin Neurol* 2018; **147:** 343-354.
58. Chabrat A, Brisson G, Doucet-Beaupré H, Salesse C, Schaan Profes M, Dovonou A *et al.* Transcriptional repression of Plxnc1 by Lmx1a and Lmx1b directs topographic dopaminergic circuit formation. *Nat Commun* 2017; **8**(1)**:** 933.
59. Liang Y, Zheng D, Peng S, Lin D, Jing X, Zeng Z *et al.* Rifampicin attenuates rotenone-treated microglia inflammation via improving lysosomal function. *Toxicol In Vitro* 2019; **63:** 104690.
60. Hoffman JL, Faccidomo S, Kim M, Taylor SM, Agoglia AE, May AM *et al.* Alcohol drinking exacerbates neural and behavioral pathology in the 3xTg-AD mouse model of Alzheimer's disease. *Int Rev Neurobiol* 2019; **148:** 169-230.

61. Huang W, Kabbani N, Brannan TK, Lin MK, Theiss MM, Hamilton JF *et al.* Association of a Functional Polymorphism in the. *J Neurotrauma* 2019; **36**(21)**:** 3026-3033.
62. Emmert AS, Iwasawa E, Shula C, Schultz P, Lindquist D, Dunn RS *et al.* Impaired neural differentiation and glymphatic CSF flow in the. *Dis Model Mech* 2019; **12**(11).
63. Forcella M, Lau P, Oldani M, Melchioretto P, Bogni A, Gribaldo L *et al.* Neuronal specific and non-specific responses to cadmium possibly involved in neurodegeneration: A toxicogenomics study in a human neuronal cell model. *Neurotoxicology* 2019; **76:** 162-173.
64. Rincon A, Paez-Rojas P, Suárez-Obando F. 8q22.2q22.3 Microdeletion Syndrome Associated with Hearing Loss and Intractable Epilepsy. *Case Rep Genet* 2019; **2019:** 7608348.
65. Gualandi F, Sette E, Fortunato F, Bigoni S, De Grandis D, Scotton C *et al.* Report of a novel ATP7A mutation causing distal motor neuropathy. *Neuromuscul Disord* 2019; **29**(10)**:** 776-785.
66. Sun XY, Zheng T, Yang X, Liu L, Gao SS, Xu HB *et al.* HDAC2 hyperexpression alters hippocampal neuronal transcription and microglial activity in neuroinflammation-induced cognitive dysfunction. *J Neuroinflammation* 2019; **16**(1)**:** 249.
67. Bertini V, Valetto A, Azzarà A, Legitimo A, Saggese G, Consolini R *et al.* A Case of 22q11 Deletion Syndrome (22q11DS) with a Panayiotopoulos Epileptic Pattern: Are Additional Copy-Number Variations a Possible Second Hit in Modulating the 22q11DS Phenotype? *Front Pediatr* 2017; **5:** 48.
68. Ding XF, Wang HJ, Qian L, Hu ZY, Feng SF, Wu Y *et al.* Cpne5 is Involved in Regulating Rodent Anxiety Level. *CNS Neurosci Ther* 2017; **23**(3)**:** 266-268.
69. Ingallinesi M, Galet B, Pegon J, Faucon Biguet N, Do Thi A, Millan MJ *et al.* Knock-Down of GPR88 in the Dorsal Striatum Alters the Response of Medium Spiny Neurons to the Loss of Dopamine Input and L-3-4-Dyhydroxyphenylalanine. *Front Pharmacol* 2019; **10:** 1233.
70. Uliana V, Bonatti F, Zanatta V, Mozzoni P, Martorana D, Percesepe A. Spectrum of X-linked intellectual disabilities and psychiatric symptoms in a family harbouring a Xp22.12 microduplication encompassing the. *J Genet* 2019; **98**.
71. Ikeda M, Takahashi A, Kamatani Y, Momozawa Y, Saito T, Kondo K *et al.* Genome-Wide Association Study Detected Novel Susceptibility Genes for Schizophrenia and Shared Trans-Populations/Diseases Genetic Effect. *Schizophr Bull* 2019; **45**(4)**:** 824-834.
72. Littlejohn BP, Price DM, Neuendorff DA, Carroll JA, Vann RC, Riggs PK *et al.* Influence of prenatal transportation stress-induced differential DNA methylation on the physiological control of behavior and stress response in suckling Brahman bull calves. *J Anim Sci* 2019.

73. Reuter MS, Tawamie H, Buchert R, Hosny Gebril O, Froukh T, Thiel C *et al.* Diagnostic Yield and Novel Candidate Genes by Exome Sequencing in 152 Consanguineous Families With Neurodevelopmental Disorders. *JAMA Psychiatry* 2017; **74**(3)**:** 293-299.
74. Verkerk AJMH, Zeidler S, Breedveld G, Overbeek L, Huigh D, Koster L *et al.* CXorf56, a dendritic neuronal protein, identified as a new candidate gene for X-linked intellectual disability. *Eur J Hum Genet* 2018; **26**(4)**:** 552-560.
75. Bento CF, Ashkenazi A, Jimenez-Sanchez M, Rubinsztein DC. The Parkinson's disease-associated genes ATP13A2 and SYT11 regulate autophagy via a common pathway. *Nat Commun* 2016; **7:** 11803.
76. Umeda K, Negishi M, Katoh H. RasGRF1 mediates brain-derived neurotrophic factor-induced axonal growth in primary cultured cortical neurons. *Biochem Biophys Rep* 2019; **17:** 56-64.
77. Monavarfeshani A, Knill CN, Sabbagh U, Su J, Fox MA. Region- and Cell-Specific Expression of Transmembrane Collagens in Mouse Brain. *Front Integr Neurosci* 2017; **11:** 20.
78. Giralt A, Brito V, Pardo M, Rubio SE, Marion-Poll L, Martín-Ibáñez R *et al.* Helios modulates the maturation of a CA1 neuronal subpopulation required for spatial memory formation. *Exp Neurol* 2020; **323:** 113095.
79. Guo D, Peng Y, Wang L, Sun X, Wang X, Liang C *et al.* Autism-like social deficit generated by Dock4 deficiency is rescued by restoration of Rac1 activity and NMDA receptor function. *Mol Psychiatry* 2019.
80. Laugel V. Cockayne syndrome: the expanding clinical and mutational spectrum. *Mech Ageing Dev* 2013; **134**(5-6)**:** 161-170.
81. Willett RT, Bayin NS, Lee AS, Krishnamurthy A, Wojcinski A, Lao Z *et al.* Cerebellar nuclei excitatory neurons regulate developmental scaling of presynaptic Purkinje cell number and organ growth. *Elife* 2019; **8**.
82. Li YQ, Koritzinsky M, Wong CS. Metabolic Regulation of Hippocampal Neuroprogenitor Apoptosis After Irradiation. *J Neuropathol Exp Neurol* 2019.
83. Mao X, Gu X, Lu W. GSG1L regulates the strength of AMPA receptor-mediated synaptic transmission but not AMPA receptor kinetics in hippocampal dentate granule neurons. *J Neurophysiol* 2017; **117**(1)**:** 28-35.
84. de Fuenmayor-Fernández de la Hoz CP, Hernández-Laín A, Olivé M, Sánchez-Calvín MT, Gonzalo-Martínez JF, Domínguez-González C. Adult-onset distal spinal muscular atrophy: a new phenotype associated with KIF5A mutations. *Brain* 2019; **142**(12)**:** e66.
85. Chen S, Zhu X, Niu W, Yao G, Kong L, He M *et al.* Regulatory Role of lncRNA NONHSAT089447 in the Dopamine Signaling Pathway in Schizophrenic Patients. *Med Sci Monit* 2019; **25:** 4322-4332.
86. Guan F, Zhang T, Liu X, Han W, Lin H, Li L *et al.* Evaluation of voltage-dependent calcium channel γ gene families identified several novel potential susceptible genes to schizophrenia. *Sci Rep* 2016; **6:** 24914.

87. Labbé C, Ogaki K, Lorenzo-Betancor O, Carrasquillo MM, Heckman MG, McCarthy A *et al.* Exonic Re-Sequencing of the Chromosome 2q24.3 Parkinson's Disease Locus. *PLoS One* 2015; **10**(6)**:** e0128586.
88. Ito Y, Carss KJ, Duarte ST, Hartley T, Keren B, Kurian MA *et al.* De Novo Truncating Mutations in WASF1 Cause Intellectual Disability with Seizures. *Am J Hum Genet* 2018; **103**(1)**:** 144-153.
89. Codina-Solà M, Rodríguez-Santiago B, Homs A, Santoyo J, Rigau M, Aznar-Laín G *et al.* Integrated analysis of whole-exome sequencing and transcriptome profiling in males with autism spectrum disorders. *Mol Autism* 2015; **6:** 21.
90. Brereton E, Fassi E, Araujo GC, Dodd J, Telegrafi A, Pathak SJ *et al.* Mutations in the PH Domain of DNM1 are associated with a nonepileptic phenotype characterized by developmental delay and neurobehavioral abnormalities. *Mol Genet Genomic Med* 2018; **6**(2)**:** 294-300.
91. Helmfors L, Bergkvist L, Brorsson AC. Serum Amyloid P Component Ameliorates Neurological Damage Caused by Expressing a Lysozyme Variant in the Central Nervous System of Drosophila melanogaster. *PLoS One* 2016; **11**(7)**:** e0159294.
92. Huo J, Xu S, Lam KP. FAIM: An Antagonist of Fas-Killing and Beyond. *Cells* 2019; **8**(6).
93. Prekop HT, Kroiss A, Rook V, Zagoraiou L, Jessell TM, Fernandes C *et al.* Sox14 Is Required for a Specific Subset of Cerebello-Olivary Projections. *J Neurosci* 2018; **38**(44)**:** 9539-9550.
94. Schulten HJ, Hussein D, Al-Adwani F, Karim S, Al-Maghrabi J, Al-Sharif M *et al.* Microarray Expression Data Identify DCC as a Candidate Gene for Early Meningioma Progression. *PLoS One* 2016; **11**(4)**:** e0153681.
95. Li S, Zhao H, Huang R, He L, Tian C, Huang H *et al.* Generation of iPSC line (GIBHi001-A) from a patient with autism spectrum disorder. *Stem Cell Res* 2019; **40:** 101571.
96. Wang YY, Shen D, Zhao LJ, Zeng N, Hu TH. Sting is a critical regulator of spinal cord injury by regulating microglial inflammation via interacting with TBK1 in mice. *Biochem Biophys Res Commun* 2019; **517**(4)**:** 741-748.
97. Bando T, Morikawa Y, Hisaoka T, Komori T, Miyajima A, Senba E. Dynamic expression pattern of leucine-rich repeat neuronal protein 4 in the mouse dorsal root ganglia during development. *Neurosci Lett* 2013; **548:** 73-78.
98. Murthy MN, Blauwendraat C, Guelfi S, Hardy J, Lewis PA, Trabzuni D *et al.* Increased brain expression of GPNMB is associated with genome wide significant risk for Parkinson's disease on chromosome 7p15.3. *Neurogenetics* 2017; **18**(3)**:** 121-133.
99. Gerhard DM, Pothula S, Liu RJ, Wu M, Li XY, Girgenti MJ *et al.* GABA interneurons are the cellular trigger for ketamine's rapid antidepressant actions. *J Clin Invest* 2019.

100. Rea G, Tirupathi S, Williams J, Clouston P, Morrison PJ. Infantile Onset of Spinocerebellar Ataxia Type 5 (SCA-5) in a 6 Month Old with Ataxic Cerebral Palsy. *Cerebellum* 2019.
101. De Toma I, Ortega M, Aloy P, Sabidó E, Dierssen M. DYRK1A Overexpression Alters Cognition and Neural-Related Proteomic Pathways in the Hippocampus That Are Rescued by Green Tea Extract and/or Environmental Enrichment. *Front Mol Neurosci* 2019; **12:** 272.
102. Wang M, Chen J, He K, Wang Q, Li Z, Shen J *et al.* The NVL gene confers risk for both major depressive disorder and schizophrenia in the Han Chinese population. *Prog Neuropsychopharmacol Biol Psychiatry* 2015; **62:** 7-13.
103. Ge X, Zhang DM, Li MM, Zhang Y, Zhu XY, Zhou Y *et al.* Microglial LOX-1/MAPKs/NF-κB positive loop promotes the vicious cycle of neuroinflammation and neural injury. *Int Immunopharmacol* 2019; **70:** 187-200.
