# Supplementary material for: Modeling alcohol-induced neurotoxicity using human induced pluripotent stem cell-derived three-dimensional cerebral organoids
Source: Transl Psychiatry. 2020 Oct 13;10:347. doi: 10.1038/s41398-020-01029-4 (PMC7553959; doi:10.1038/s41398-020-01029-4)
Supplement: Supplementary file 5 — Supplemental Table 3 [file 41398_2020_1029_MOESM5_ESM.pdf]

Supplemental table 3. Abnormally expressed genes related to 37 diseases and function signaling shown in Figure 6A-a

Category	P-value	Molecules
Organismal Injury and Abnormalities	5.17E-13-9.06E-03	AASDHPPT,ACSL4,ACTBL2,ADAM21,ALG10B,ALKBH8,ALX3,ANK3,ANKRD33B,APCS,ARFGEF1,ATP6V0A1,ATP7A,B3GALT1,BROX,C11orf87,C11orf94,C5orf47,C5orf63,CABYR,CACNG6,CBWD1,CCDC117,CCN2,CCSER1,CDCP2,CER1,CHD5,CHIC1,CHRFAM7A,CLIP1,CNTN1,COL25A1,CPNE5,CSRNP3,CXorf56,DDN,DDX42,DENND2A,DGKD,DISP2,DNM1,DOC2B,DOCK4,DPYSL2,DRD1,DRD5,DYRK1A,EFR3B,EN2,EP400,EPHA7,ERCC6,FAIM,FAM149A,FAM234B,FANCD2,FAT4,FCGR1B,FRMD5,GABRA2,GABRG3,GAD1,GAN,GGA3,GPD1L,GPR88,GRAP2,GREM2,GSG1L,GUCY1A2,HDAC2,HELQ,HOXC13,HSD11B2,HSDL1,IKZF5,IST1,KBTBD2,KCNB2,KCNC1,KCNS2,KCNV1,KIF5A,KIRREL3,KRTAP4-11,KRTAP9-7,L1CAM,LGALS12,LRRC8B,LRRN4,MADD,MAFA,MAPK8IP2,MAPK8IP3,MARCHF11,MAS1,MBD3L1,MEF2A,MEF2C,MYCBP2,NAB1,NCOA1,NFS1,NUP42,NVL,OLR1,OR2A12,PAGE4,PAOX,PCDHB11,PCDHB8,PDGFC,PDXDC1,PER2,PEX5L,PLXNC1,POLN,PPFIA3,PRKAA2,PROCA1,PRR14L,PRR35,PRRT4,PTPDC1,RASGEF1B,RASGRF1,RGPD4 (includes others),RIMKLA,RORB,RPS6KA3,RXFP1,SASS6,SCML1,SEMA4C,SENP5,SEPTIN12,SFMBT2,SHPRH,SLC16A14,SLC3A1,SLITRK1,SNRK,SOX14,SPHKAP,SPTB,SPTBN2,SRD5A1,SS18L1,SSH2,STK10,STRN3,TBC1D2B,TBC1D32,TBX1,TERF2,TLE1,TMCO6,TMEM173,TMEM196,TMEM245,TRAPPC11,TRMT9B,TTPAL,TUSC1,UBD,USPL1,VSNL1,WASF1,WNT7A,ZBTB18,ZC3H4,ZKSCAN2,ZNF266,ZNF350,ZNF362,ZNF770,ZNF852,ZXDA
Cell Morphology	2.98E-08-8.48E-03	ANK3,ARFGEF1,ATP7A,CCN2,CNTN1,COL25A1,DNM1,DPYSL2,DRD1,DRD5,DYRK1A,EP400,EPHA7,ERCC6,FAM234B,GAD1,GAN,HDAC2,KIF5A,KIRREL3,L1CAM,MADD,MAPK8IP2,MAPK8IP3,MEF2A,MEF2C,MYCBP2,NAB1,NCOA1,PLXNC1,PPFIA3,PRKAA2,PTPDC1,RASGRF1,RPS6KA3,SLITRK1,SPTB,SPTBN2,SS18L1,STRN3,TBC1D32,TMCO6,USPL1,WASF1,WNT7A,ZBTB18
Cellular Development	2.98E-08-8.05E-03	ACSL4,ANK3,ARFGEF1,ATP7A,CCN2,CNTN1,COL25A1,DOCK4,DPYSL2,DRD1,DYRK1A,EN2,EPHA7,FANCD2,HDAC2,KIRREL3,L1CAM,MAPK8IP2,MAPK8IP3,MEF2A,MEF2C,MYCBP2,NAB1,NCOA1,PCDHB11,PCDHB8,PDGFC,PRKAA2,RASGRF1,RORB,RPS6KA3,SEPTIN12,SLITRK1,SPTBN2,SS18L1,TBX1,TMEM173,WASF1,WNT7A,ZBTB18
Cellular Growth and Proliferation	2.98E-08-8.05E-03	ANK3,ARFGEF1,ATP7A,CCN2,CNTN1,COL25A1,DOCK4,DPYSL2,DRD1,DYRK1A,EN2,EPHA7,HDAC2,KIRREL3,L1CAM,MAPK8IP2,MAPK8IP3,MEF2A,MEF2C,MYCBP2,PCDHB11,PCDHB8,PDGFC,PRKAA2,RASGRF1,RPS6KA3,SLITRK1,SPTBN2,SS18L1,TMEM173,UBD,WASF1,WNT7A,ZBTB18

Nervous System Development and Function	2.98E-08-8.75E-03	ACSL4,ANK3,ARFGEF1,ATP7A,CCN2,CNTN1,COL25A1,DNM1,DOC2B,DOCK4,DPYSL2,DRD1,DRD5,DYRK1A,EN2,EPHA7,ERCC6,FANCD2,FAT4,GAD1,GAN,GPR88,GUCY1A2,HDAC2,HSD11B2,KCNC1,KIF5A,KIRREL3,L1CAM,MADD,MAPK8IP2,MAPK8IP3,MAS1,MEF2A,MEF2C,MYCBP2,NAB1,NCOA1,PCDHB11,PCDHB8,PDGFC,PER2,PEX5L,PLXNC1,PPFIA3,PRKAA2,RASGRF1,RORB,RPS6KA3,SEMA4C,SLITRK1,SPTB,SPTBN2,SS18L1,TBX1,VSNL1,WASF1,WNT7A,ZBTB18
Organismal Development	2.98E-08-9.06E-03	ALX3,ANK3,ARFGEF1,ATP7A,CCN2,CER1,CNTN1,COL25A1,DPYSL2,DRD1,DYRK1A,EN2,EPHA7,ERCC6,FANCD2,FAT4,GABRA2,GABRG3,GAD1,GREM2,HDAC2,HOXC13,HSD11B2,KIRREL3,L1CAM,MAPK8IP2,MAPK8IP3,MEF2A,MEF2C,MYCBP2,NCOA1,PDGFC,PER2,PRKAA2,RASGRF1,RORB,RPS6KA3,RXFP1,SEMA4C,SEPTIN12,SLITRK1,SPTB,SPTBN2,SS18L1,TBC1D32,TBX1,TLE1,WASF1,WNT7A,ZBTB18
Tissue Development	2.98E-08-9.06E-03	ACSL4,ANK3,APCS,ARFGEF1,ATP7A,CCN2,CNTN1,COL25A1,DOCK4,DPYSL2,DRD1,DYRK1A,EN2,EPHA7,ERCC6,FANCD2,FAT4,HDAC2,HOXC13,HSD11B2,KIRREL3,L1CAM,MAPK8IP2,MAPK8IP3,MEF2A,MEF2C,MYCBP2,NAB1,NCOA1,PCDHB11,PCDHB8,PDGFC,PER2,PRKAA2,RASGRF1,RORB,RPS6KA3,RXFP1,SEMA4C,SLITRK1,SPTBN2,SS18L1,TBX1,WASF1,WNT7A,ZBTB18
Neurological Disease	9.45E-08-8.83E-03	ACSL4,ANK3,ATP7A,CCN2,CDCP2,CNTN1,COL25A1,CPNE5,CXorf56,DDX42,DNM1,DPYSL2,DRD1,DRD5,DYRK1A,EN2,EPHA7,ERCC6,FAM149A,FANCD2,FCGR1B,GABRA2,GABRG3,GAD1,GAN,GGA3,GPR88,HDAC2,KCNB2,KCNC1,KCNS2,KCNV1,KIF5A,KIRREL3,L1CAM,LRRC8B,LRRN4,MADD,MAPK8IP2,MAPK8IP3,MEF2C,MYCBP2,NAB1,NCOA1,NFS1,OLR1,PDGFC,PER2,PEX5L,PLXNC1,PRKAA2,RASGRF1,RORB,RPS6KA3,SLITRK1,SNRK,SPTBN2,SRD5A1,SS18L1,STRN3,TBX1,VSNL1,WASF1,ZBTB18
Tissue Morphology	9.45E-08-8.95E-03	ANK3,APCS,ATP7A,CCN2,CNTN1,COL25A1,DNM1,DPYSL2,DRD1,DYRK1A,EN2,EP400,EPHA7,ERCC6,FANCD2,GAD1,GAN,HDAC2,HSD11B2,KIF5A,L1CAM,MADD,MAPK8IP2,MAPK8IP3,MEF2A,MEF2C,MYCBP2,NCOA1,PDGFC,PLXNC1,PRKAA2,RASGRF1,RPS6KA3,SLITRK1,SPTB,SPTBN2,TERF2,WASF1,WNT7A,ZBTB18
Cellular Assembly and Organization	3.63E-07-8.5E-03	ANK3,APCS,ARFGEF1,ATP7A,CCN2,CLIP1,CNTN1,COL25A1,DNM1,DOCK4,DPYSL2,DRD1,DYRK1A,EPHA7,ERCC6,FANCD2,FAT4,GAN,HDAC2,KIF5A,KIRREL3,L1CAM,MADD,MAPK8IP2,MAPK8IP3,MEF2A,MEF2C,MYCBP2,OLR1,PCDHB11,PCDHB8,PPFIA3,PRKAA2,PTPDC1,RASGRF1,RPS6KA3,SLITRK1,SPTB,SPTBN2,SS18L1,SSH2,STRN3,TBC1D32,TERF2,TRAPPC11,WASF1,WNT7A

Cellular Function and Maintenance	3.63E-07-8.5E-03	ANK3,ARFGEF1,ATP7A,CCN2,CLIP1,CNTN1,COL25A1,DNM1,DOCK4,DPYSL2,DRD1,DYRK1A,EPHA7,ERCC6,GAN,HDAC2,KIF5A,KIRREL3,L1CAM,MAPK8IP2,MAPK8IP3,MEF2A,MEF2C,MYCBP2,PCDHB11,PCDHB8,PRKAA2,PTPDC1,RASGRF1,RPS6KA3,SLITRK1,SPTBN2,SS18L1,SSH2,TBC1D32,TRAPPC11,WASF1,WNT7A
Developmental Disorder	2.43E-06-8.05E-03	ACSL4,ALX3,ANK3,ATP7A,CCN2,CNTN1,COL25A1,CXorf56,DNM1,DRD1,DRD5,DYRK1A,EN2,ERCC6,FANCD2,FAT4,FCGR1B,GABRA2,GABRG3,GAD1,HOXC13,KCNS2,KIRREL3,L1CAM,MAPK8IP3,MEF2C,NCOA1,PDGFC,RPS6KA3,RXFP1,SASS6,SEPTIN12,SPTB,TBX1,TRAPPC11,WNT7A,ZBTB18
Cell-To-Cell Signaling and Interaction	5.47E-06-8.48E-03	ANK3,CCN2,CNTN1,DNM1,DOC2B,DPYSL2,DRD1,DRD5,DYRK1A,EPHA7,FAT4,GAD1,GPR88,GUCY1A2,HDAC2,HSD11B2,KCNC1,KIF5A,KIRREL3,L1CAM,MADD,MAPK8IP2,MAS1,MEF2A,MEF2C,MYCBP2,OLR1,PCDHB11,PCDHB8,PPFIA3,PRKAA2,RASGRF1,RPS6KA3,SLITRK1,SPTBN2,WASF1,WNT7A
Hereditary Disorder	1.48E-05-8.05E-03	ACSL4,ANK3,ATP7A,CCN2,CNTN1,COL25A1,CXorf56,DNM1,DRD1,DRD5,DYRK1A,EN2,ERCC6,FANCD2,FAT4,FCGR1B,GABRA2,GABRG3,GAD1,GAN,GPD1L,GPR88,GREM2,HOXC13,HSD11B2,KCNC1,KIF5A,KIRREL3,L1CAM,MEF2A,MEF2C,OLR1,PER2,RORB,RPS6KA3,SASS6,SEPTIN12,SLITRK1,SPTB,SPTBN2,TBX1,TMEM173,TRAPPC11,VSNL1,WNT7A,ZBTB18
Psychological Disorders	1.48E-05-8.05E-03	ANK3,CCN2,COL25A1,DNM1,DPYSL2,DRD1,DRD5,DYRK1A,EN2,FAM149A,FCGR1B,GABRA2,GABRG3,GAD1,GGA3,KCNB2,KCNS2,KIF5A,LRRC8B,MEF2C,NFS1,OLR1,PDGFC,PER2,SLITRK1,SNRK,SRD5A1,STRN3,VSNL1,WASF1
Organ Morphology	3.26E-05-9.06E-03	ANK3,CCN2,CNTN1,DRD1,DYRK1A,EN2,EPHA7,ERCC6,HDAC2,HOXC13,HSD11B2,L1CAM,MAPK8IP2,MAPK8IP3,MEF2A,MEF2C,MYCBP2,NCOA1,PRKAA2,RPS6KA3,RXFP1,SPTBN2,SS18L1,TBX1,WASF1,WNT7A,ZBTB18
Organismal Survival	3.42E-05-4.78E-04	ANK3,APCS,ATP7A,CCN2,CNTN1,COL25A1,CSRNP3,DGKD,DNM1,DRD1,DRD5,DYRK1A,EP400,ERCC6,FAIM,FANCD2,FAT4,GABRA2,GAD1,GAN,GPD1L,HDAC2,HOXC13,HSD11B2,KBTBD2,KCNC1,KIF5A,L1CAM,MADD,MAPK8IP2,MAPK8IP3,MEF2A,MEF2C,MYCBP2,NAB1,NCOA1,PDGFC,PER2,PRKAA2,RGPD4 (includes others),SLITRK1,SNRK,SS18L1,TBX1,TERF2,TMEM173,UBD,WASF1,WNT7A,ZBTB18
Cellular Compromise	6.45E-05-8.05E-03	ANK3,APCS,COL25A1,DNM1,KIF5A
Post-Translational Modification	6.45E-05-8.05E-03	APCS,COL25A1
Protein Degradation	6.45E-05-8.05E-03	APCS,COL25A1

Protein Synthesis	6.45E-05-8.05E-03	AKAIN1,ANK3,APCS,COL25A1,DDX42,DOC2B,EFR3B,IST1,KCNB2,LGALS12,MAPK8IP3,MYCBP2,NCOA1,PER2,PRKAA2,RPS6KA3
Behavior	1.2E-04-8.75E-03	ANK3,ATP7A,CCN2,CNTN1,COL25A1,DNM1,DRD1,DRD5,DYRK1A,EN2,ERCC6,GABRA2,GAD1,GAN,GPR88,HDAC2,HSD11B2,KCNC1,L1CAM,LRRN4,MAPK8IP2,NCOA1,PER2,PEX5L,PRKAA2,RASGRF1,RORB,RPS6KA3,TBX1,WASF1
Cellular Movement	1.92E-04-8.05E-03	ANK3,CCN2,CNTN1,DNM1,DPYSL2,DRD1,GAD1,KIRREL3,L1CAM,MAPK8IP3,MEF2C,MYCBP2,NCOA1,PLXNC1,PPFIA3,RPS6KA3,SPTB,SPTBN2,ZBTB18
Cell Death and Survival	3.83E-04-8.05E-03	COL25A1,EP400,KIF5A,NCOA1,OLR1,PDGFC,SPTB,UBD,WNT7A
Embryonic Development	3.83E-04-9.06E-03	ALX3,ANK3,CCN2,CER1,CNTN1,COL25A1,DNM1,DPYSL2,DRD1,DYRK1A,EN2,EPHA7,ERCC6,FANCD2,FAT4,GABRA2,GABRG3,GAD1,HDAC2,HOXC13,KIRREL3,L1CAM,MAPK8IP3,MEF2C,NCOA1,PDGFC,PER2,PRKAA2,RORB,RPS6KA3,RXFP1,SEMA4C,SEPTIN12,SPTBN2,TBC1D32,TBX1,TLE1,WASF1,WNT7A,ZBTB18
Organ Development	3.83E-04-9.06E-03	ANK3,CCN2,CNTN1,DRD1,DYRK1A,EN2,EPHA7,ERCC6,FANCD2,FAT4,HDAC2,HOXC13,HSD11B2,KIRREL3,L1CAM,MAPK8IP3,MEF2C,NAB1,NCOA1,PDGFC,RORB,RXFP1,SEMA4C,SEPTIN12,TBX1,WNT7A,ZBTB18
Organismal Functions	1.06E-03-8.05E-03	ANK3,CER1,CNTN1,DNM1,DRD1,GAN,HOXC13,HSD11B2,KCNB2,KCNC1,L1CAM,NAB1,SLITRK1,TRAPPC11
Gene Expression	2.28E-03-6.93E-03	ANK3,APCS,ATP7A,CCN2,CHD5,CNTN1,CSRNP3,DDN,DRD1,DYRK1A,EN2,ERCC6,HDAC2,HOXC13,IKZF5,L1CAM,MAFA,MAPK8IP3,MBD3L1,MEF2A,MEF2C,NAB1,NCOA1,PER2,PRKAA2,RASGRF1,RORB,RPS6KA3,RXFP1,SFMBT2,SLC3A1,SS18L1,STRN3,TBX1,TERF2,TLE1,TMEM173,USPL1,WNT7A,ZBTB18,ZNF350,ZXDA
Molecular Transport	2.46E-03-9.06E-03	AKAIN1,ANK3,ATP7A,CNTN1,DDX42,DOC2B,EFR3B,ERCC6,FANCD2,GPD1L,IST1,KCNB2,KCNC1,KCNS2,KCNV1,MAPK8IP3,MYCBP2,SLC3A1
Protein Trafficking	2.46E-03-2.46E-03	AKAIN1,ANK3,DDX42,DOC2B,EFR3B,IST1,KCNB2,MAPK8IP3,MYCBP2
Cell Signaling	2.78E-03-4.34E-03	CCN2,DRD1,DRD5,ERCC6,MAPK8IP2,MAPK8IP3,MEF2A,MEF2C,RASGRF1,SPTB,SPTBN2,TBX1,TLE1,UBD,WNT7A
Cell Cycle	3.38E-03-8.05E-03	DNM1,DYRK1A,EP400,FANCD2,GRAP2,HDAC2,RPS6KA3,SSH2,TERF2
DNA Replication, Recombination, and Repair	3.38E-03-8.05E-03	FANCD2,TBX1,TERF2

Lipid Metabolism	3.79E-03-8.05E-03	ACSL4,B3GALT1,CLIP1,HSD11B2,MAS1,SRD5A1
Small Molecule Biochemistry	3.79E-03-8.05E-03	ACSL4,B3GALT1,CLIP1,HSD11B2,MAS1,SLC3A1,SRD5A1
Metabolic Disease	5.15E-03-8.05E-03	APCS,ATP7A,CCN2,COL25A1,DPYSL2,DRD1,DRD5,DYRK1A,ERCC6,FANCD2,FCGR1B,GABRA2,GABRG3,GGA3,HSD11B2,NFS1,OLR1,STRN3,TMEM173,WASF1
Energy Production	8.05E-03-8.05E-03	PAOX
